# Supply Chain Factors Contributing to Improved Material Flow Indicators but Increased Carbon Footprint

**DOI:** 10.1021/acs.est.3c00859

**Published:** 2023-08-17

**Authors:** Sho Hata, Keisuke Nansai, Kenichi Nakajima

**Affiliations:** † Material Cycles Division, National Institute for Environmental Studies, 16-2 Onogawa, Tsukuba, Ibaraki 305-8506, Japan; ‡ Graduate School of Frontier Sciences, The University of Tokyo, 5-1-5 Kashiwanoha, Kashiwa, Chiba 277-8563, Japan

**Keywords:** input−output analysis, material flow indicator, structural decomposition analysis, capital endogenizing method

## Abstract

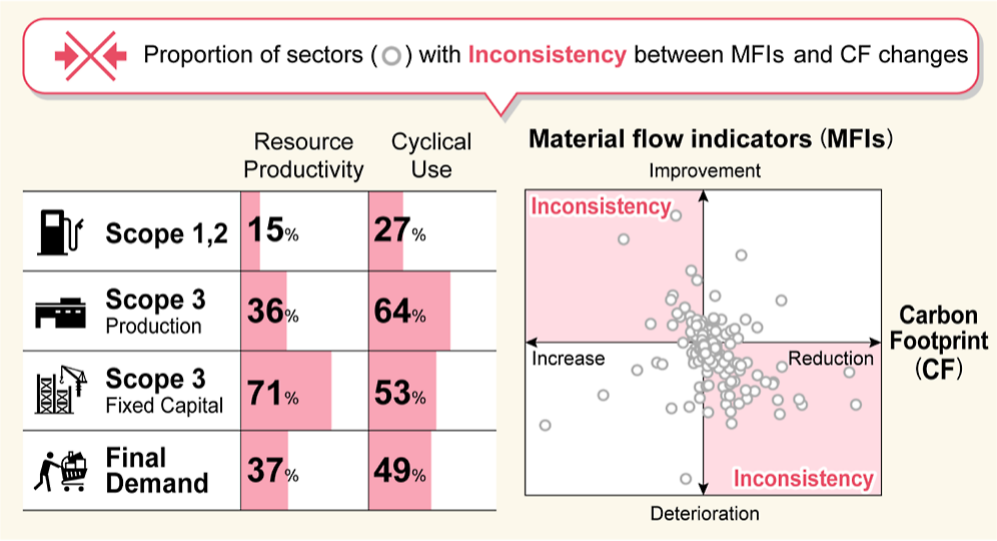

Improvements in four material flow indicators (MFIs) have helped facilitate Japan’s transition to a sound material-cycle society. However, the economic and technological factors that have affected these MFIs have not been identified previously. Moreover, it is unclear whether the improvements in the MFIs have contributed to Japan’s progress toward carbon mitigation. In this study, we quantified the contribution of the factors in the capital-embodied supply chain to changes in the MFIs at the national and sector levels. We also examined the consistency of MFI improvements with carbon footprint reduction. Our results show that, in many sectors, structural changes in the supply chain improved two of the MFIs (resource productivity and material circularity) but increased the carbon footprint of the sector. To address this conflict, producers need to manage their supply chains based on an understanding of the nexus between material consumption and carbon emissions, paying particular attention to supply chains associated with capital formation.

## Abstract 2

Material management toward carbon neutrality requires a comprehensive assessment of both material flows and carbon emissions to ensure that the policies promoting material efficiency align with the efforts to combat climate change.

## 1 Introduction

The large-scale consumption of natural resources has historically been accepted as the cost of economic growth and personal well-being.^1^ However, consuming natural resources at the current pace is unsustainable, and the need to reduce resource consumption through more efficient use has become increasingly apparent.^2^ The state of material utilization in a particular country, including inflow, outflow, and cyclical use, has been tracked with economy-wide material flow accounting (EW-MFA).^3−6^ Raw material consumption and material footprint (MF),^7,8^ two consumption-based indicators, have also been used to estimate the direct and indirect material use of a country through international trade. For instance, the countries with the largest per capita material footprints are Australia, Japan, and the United States, each of which exceeded 25 t/cap/year as of 2008.^7^ The material footprint of the world increased significantly until 2014, driven by emerging economies in the Asia-Pacific region, including China, but has since plateaued.^8^

Among the countries with the largest per capita material footprints, Japan is the only country that has seen a downward trend in total material inflows,^7^ which fell from 2.1 billion tons in 2000 to 1.5 billion tons in 2018.^9^ This decline is largely attributed to the fact that, in 2006, Japan adopted four material flow indicators in its fundamental plan for a sound material-cycle society (hereafter, we refer to these four material flow indicators as MFIs) and has set numerical targets every 5 years in an effort to curb material use.^10^ The adopted MFIs include (1) resource productivity (RP), which is GDP per total input of natural resources and imported products, i.e., domestic material input (DMI); (2) final disposal (FD), the amount of landfilled waste; (3) the cyclical use rate of inflow (CU_in_), which measures the amount of cyclical use per total input of material [natural resources, imported products, and cyclical use (CU)], and the cyclical use rate of outflow (CU_out_), which is cyclical use per amount of generated waste.^9,10^ Improvements in these indicators have contributed to a reduction in Japan’s per capita material inflow from 17 t in 2000 to 12 t in 2018.^9^

The use of materials is a major trigger for greenhouse gas (GHG) emissions^11,12^ or carbon footprint. Indeed, it has been reported that GHG emissions from resource production accounted for 25% of global GHG emissions in 2015.^13^ Notably, large amounts of resources are input for fixed capital formation,^13−15^ and these also induce substantial GHG emissions in processes that extend from mining to material production.^16^ This implies that changes in a country’s material flows within the country are intimately linked to the dynamics of GHG emissions. However, this link has not been taken into account in the setting of the Japan’s MFI targets. Given this disconnect, it is questionable whether further improvements in MFIs will actually contribute to the decarbonization of Japan, a country that has pledged in the Paris Agreement to reduce GHG emissions by 46% relative to 2013 levels by 2030 and to achieve carbon neutrality by 2050.^17^

To gain insight into the association between MFIs and carbon footprint, it is vital to specify the factors that change the indicators and to understand the impact of those factors on GHG emissions. Hashimoto et al.^18^ decomposed the changes in RP in Japan from 1995 to 2002 and discovered that the structure of final demand and the impact of demand in specific sectors (construction, machinery, and services) were the most influential drivers. Tanikawa et al.^19^ decomposed the changes in RP into primary use rate, retention time, and stock productivity and underlined the importance of the stock-oriented material indicators. However, to date, no studies have identified the key drivers of improvement in each of the four official MFIs or addressed the consistency of aiming for both MFI improvement and GHG reduction.

To this end, this study formulates the four MFIs and the carbon footprint at the national and industrial sector levels using economic and technological variables and identifies the variables that most influence changes in the MFIs. It then examines the linkage between MFI improvement and changes in the carbon footprint.

## 2 Methods and Data

### 2.1 Formulation of MFIs with the Capital Endogenized Input–Output Model

Japan’s MFIs are defined as follows: RP—GDP per total input of the natural resources and imported products, i.e., domestic material input; FD—the amount of landfilled waste; CU_in_—the amount of CU per total input of material (natural resources, imported products, and CU); CU_out_—CU per amount of generated waste (GW). These MFIs can be formulated with Japan’s capital endogenized input–output (IO)^16,20^ model as follows
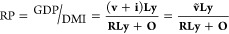
1
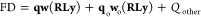
2
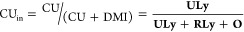
3
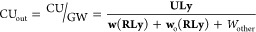
4where  is the Leontief inverse matrix. Matrix  is composed of the input coefficients including the endogenized fixed capital effects, excluding the spillover effects of imports. Matrix **I** is an identity matrix. Matrix **A** = (*a*_*ij*_) represents the input of commodity *i* into the activity of industry *j*. Matrix **B** = (*b*_*il*_), the capital formation matrix, is composed of the inputs of commodity *i* to *l* type sectors of fixed capital formation. Matrix **C** = (*c*_*lj*_), the capital utilization matrix, describes *l* types of fixed capital utilization with respect to unit production in sector *j* (see our previous work^16^ for more information regarding endogenous fixed capital formation and utilization effects). Each element *m*_*i*_ of vector **m** = (*m*_*i*_) represents the import ratio of commodity *i*. Vector **y** = (*y*_*i*_) represents the final demand for commodity *i*. For domestic demand, we exclude the spillover effect of imports using vector **m**. Vector **v** = (*v*_*j*_) shows the amount of value-added per total output in sector *j*; matrix **i** is an identity matrix.

Matrix **R** = (*r*_*kj*_) is composed of elements *r*_*kj*_, each of which represents the direct input of natural resources and imported products *k* per unit production in sector *j*; matrix **O** = (*o*_*kj*_) represents the direct consumption of natural resources and imported products *k* to sector *j* of final demand. Vector **w** = (*w*_*i*_) represents the industrial waste generation rate of sector *i*; vector **w**_o_ = (*w*_o,*i*_) represents the municipal waste generation rate of commodity *i*; vector **q** = (*q*_*i*_) represents the final disposal rate of industrial waste of sector *i*; and **q**_o_ = (*q*_*o*,*i*_) represents the final disposal rate of municipal waste of commodity *i*. Matrix **U** = (*u*_*sj*_) shows the direct input of CU, *s*, per unit production in sector *j*. *W*_other_ and *Q*_other_ represent other waste generation and other final disposal, respectively.

We calculate the capital-embodied carbon footprint (CF) as CF = **eLy** + **G**. Here, vector **e** = (*e*_*j*_), where *e*_*j*_ is the carbon emissions per unit production in sector *j*, and vector **G** = (*g*_*j*_), where *g*_*j*_ is the direct carbon emission from sector *j* of final demand.

### 2.2 Structural Decomposition Analysis of the Material Flow Indicators

Using the components of RP in eq 1, we can express the change in RP between specific terms *t* and *t* + 1 as
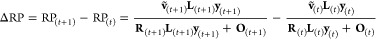
5

Here, the challenge in conducting a structural decomposition analysis (SDA) of eq 5 is that there are multiple forms of decomposition for a change in the same period. That is, for every *n* decomposition term, there are *n*! solutions and no unique solution. To address this “non-uniqueness problem”, Dietzenbacher and Los^21^ proposed computing the average of the solutions of all the decomposition forms as the solution to the SDA. The full mean value shown in eq 6 is commonly used as the SDA solution^22−26^
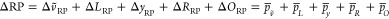
6where  represents the full mean value of SDA for driver *n*. In this study,  were decomposed as follows to identify the key change drivers for each industry
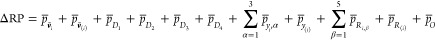
7where , , and  are the supply chain effects of sector *i*, broken down for the scope of the GHG protocol.^27^, , and  are the supply chain of direct energy inputs (Scope 1 and 2), the production supply chain of goods and services (Scope 3; production), and the supply chain utilizing fixed capital (Scope 3; fixed capital) in sector *i*, respectively.  represents the supply chain effect for all industries except sector *i*, where . By using Japan’s capital endogenized IO model,^16^ the supply chain effect of fixed capital utilization () is separated from the production effect (). The effect of sector *i* was extracted for , , and  as well. In addition,  was decomposed into five resource types (α = 1...5; biomass, fossil fuels, metals, non-metallic minerals, and imported products) and  into three final demands (β = 1...3; household consumption, other domestic consumption, and exports). See Supporting Information for more detailed descriptions of each decomposition driver and SDA of the other MFIs and CF.

According to the number of drivers, *n*, in eq 7, finding the full mean value of the SDA of this RP would require the calculation of *n* = 17! cases, effectively rendering the calculation of such a huge number of decomposed forms unrealistic. As a possible alternative, it has been shown that calculating the mean of a bipolar^21^ or mirror-image^28^ pair yields a value close to the full mean value. While these two methods may be considered as alternatives to full decomposition when the number of driving factors is large,^29−32^ it has been pointed out that they fail the factor-reversal test and are not ideal.^33^ Moreover, the original papers^21,28^ proposing these alternative methods only confirmed alternativity with the full mean value when there are relatively few decomposition terms (fewer than *n* = 5). To our knowledge, there are no examples of using these alternative means for decomposition terms greater than *n* = 15. To overcome the “non-uniqueness problem” with a large number of decomposition terms, we used the average of 100 randomly generated pairs of mirror images as an alternative solution to the full average. Details of this “random-mirror decomposition” and the SDA of MFIs other than RP are described in the Supporting Information.

### 2.3 Data Compilation

Matrices **A**, **B**, and **C** and vectors **v** and **y** are determined using the Japanese Time-series Input–Output Table (TJIOT) for 2005–2011–2015,^34^ the Japanese Input–Output Table (JIOT) for 2015 and 2011, and the table of fixed capital formation in the Supporting Information of the JIOT. We constructed the table of fixed capital formation in 2011 using the 2015 price basis by allocating the total final demand of fixed capital formation taken from the TJIOT according to the ratio of inputs to *l* types of fixed capital formation for sector *i* taken from the 2011 JIOT. See our previous work^16^ for the detailed data and methodology used to construct input coefficient matrix **A** with endogenized fixed capital. The **A**^*d*^ matrix compiled using the above data has 378 industrial sectors and 106 categories of fixed capital formation.

For matrices **R** and **U**, we obtained the data for domestically mined resources, domestic recycled materials, and imported resources and products from several trades and resource statistics (please refer to our previous work^16^ for more information on the statistics used). **R** consists of the input data for 45 resource categories (*k* = 1...45) of 44 natural resources and imported products; **U** consists of 7 types of CU materials (*s* = 1...7). For the vectors **w**, **w**_o_, **q**, and **q**_o_, we obtained the amount of generated industrial and municipal waste and the amount of final disposal wastes in each sector from the Survey Report on Industrial Waste Generation and Disposal Status.^35^ For each of the 19 types of industrial waste and 9 types of municipal waste, the waste generation rate per material input to each sector and the final disposal rate per waste volume were calculated.

For vectors **e** and **G**, we used the Embodied Energy Emissions Intensity Data for Japan Using Input–Output Tables (3EID).^36,37^ Vector **G** deals with direct emissions from household consumption and other domestic final demand produced by the combustion of automotive and heating fuels, with zero emissions from exports.

## 3 Results

### 3.1 Critical Drivers of the Improvement in the National MFIs

**Figure 1:**
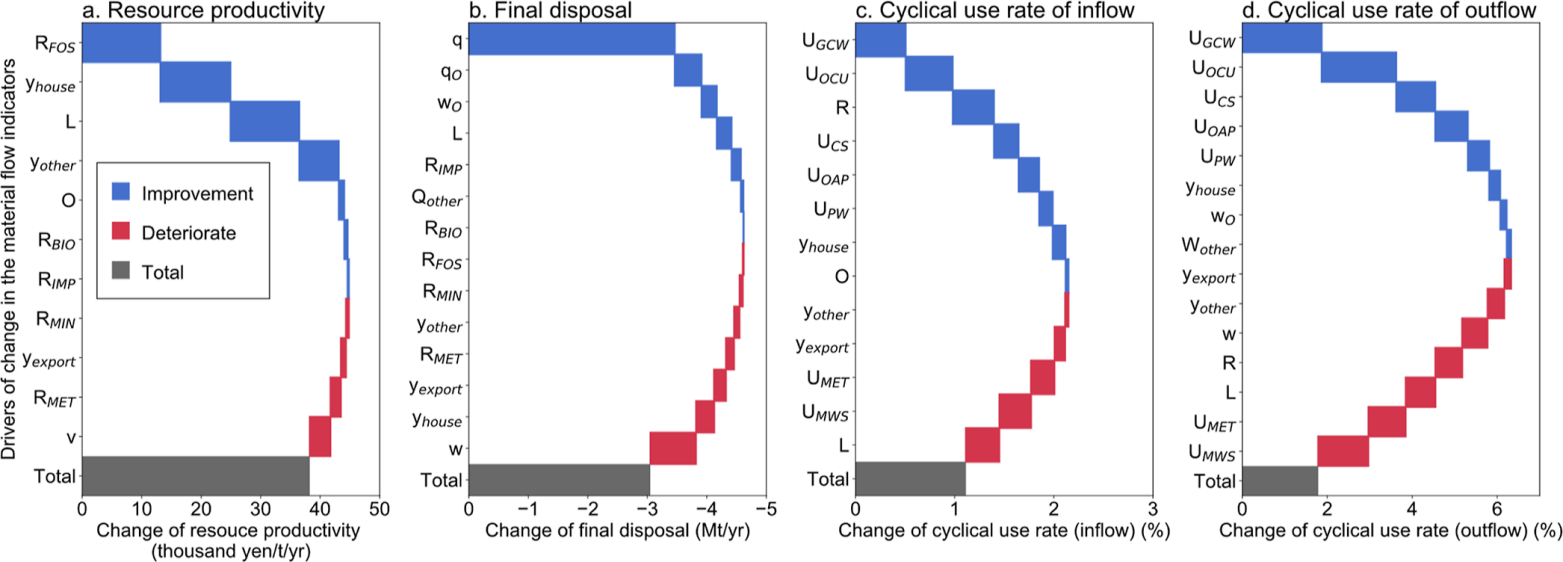
Driving forces of change in the material flow indicators. Drivers of improvement or deterioration from 2011 to 2015 for resource productivity (a), final disposal (b), cyclical use rate of inflow (c), cyclical use rate of outflow (d): *L*, supply chain structure; *v*, rate of value-added; *O*, direct resource consumption; **w**, industrial-waste generation rate; **w**_o_, municipal-waste generation rate; *W*_other_, other waste generation; **q**, final disposal rate of industrial-waste; **q**_o_, final disposal rate of municipal-waste; and *Q*_other_, other final disposal. *R* refers to the material use intensity of natural resource; *R*_BIO_, biomass; *R*_FOS_, fossil fuels; *R*_MET_, metals; *R*_MIN_, non-metallic minerals; and *R*_IMP_, imported products. *U* refers to the material use intensity of cyclical use; *U*_CS_, cinders and sludge; *U*_OAP_, oil, acid/alkali and plastic waste; *U*_PW_, paper waste; *U*_MET_, metal waste; *U*_GCW_, grass and ceramic waste; *U*_MWS_, mining waste and slug; and *U*_OCU_, other cyclical use. *y* refers to the final demand; *y*_house_, household consumption; *y*_other_, other domestic consumption; and *y*_export_, export.

The Japanese RP improved by approximately 40000yen/t/year from 2011 (389,000 yen/t/year) to 2015 (427,000 yen/t/year). The greatest drivers of the improvement were changes in the direct input of materials per unit production [material use intensity (t/million yen)] of fossil fuels, *R*_FOS_, the final demand of household consumption, *y*_house_, and the supply chain structure, *L* (Figure 1a). FD also improved, as evidenced by a reduction of 3 Mt/year from 2011 (17 Mt/year) to 2015 (14 Mt/year), with the largest driver being the final disposal rate of industrial-waste, **q**, followed by the final disposal rate of municipal waste, **q**_o_, and the municipal waste generation rate, **w**_o_ (Figure 1b). The CU indicators improved by 1% (from 15% of 2011 to 16% of 2015) for inflow and 2% (from 42% to 44%) for outflow. The main drivers of the improvement in CU_in_ were the material use intensity of glass and ceramic waste, *U*_GCW_, the material use intensity of other recycled materials, *U*_OCU_, and the material use intensity of natural resources, *R* (Figure 1c). The main drivers of the change in CU_out_ were similar to those for CU_in_; they included *U*_GCW_, *U*_OCU_, and *U*_CS_ (Figure 1d). It is apparent that the changes in MFIs at the national level do not depend on the changes in isolated factors but rather on a combination of economic and technological drivers.

Drivers *L* (supply chain structure) and *y* (final demand) are common factors for the four MFIs, but they affect each indicator differently. The change in supply chain structure, *L*, contributed to the improvement of RP and FD but had a negative effect on the CU rates (CU_in_ and CU_out_). On the other hand, the final demand of exports, *y*_export_, negatively impacted all the MFIs. The impact of material use intensity (*R* and *U*) on the MFIs differed by the type of material. Natural resources, *R*, improved RP and CU_in_, with fossil fuels (*R*_FOS_) and biomass (*R*_BIO_**)** as the main drivers. However, metals (*R*_MET_) and non-metallic (*R*_MIN_) minerals were drivers of deterioration in RP and CU rates (see Table S3 in the Supporting Information).

### 3.2 Critical Drivers of Changes in the Sectoral MFIs

**Figure 2:**
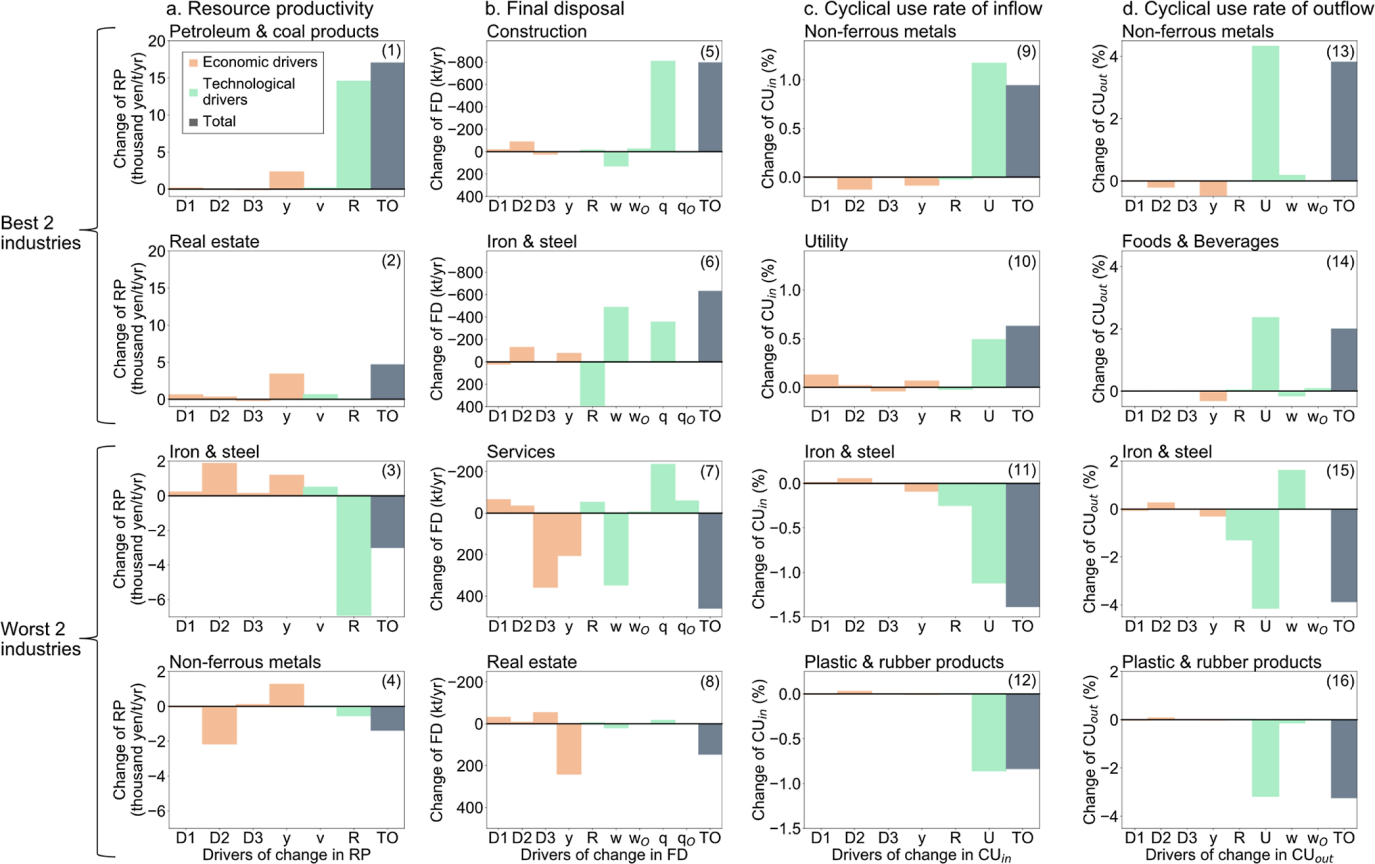
Driving forces contributing to the changes in the material flow indicators at the industry level. Two best and worst industries that improved resource productivity (a), final disposal (b), cyclical use rate of inflow (c), and cyclical use rate of outflow (d).

Although all the MFIs improved at the national level, at the industry level, there were industries whose MFIs improved and worsened (Figure 2). For simplicity, the 378 sectors were grouped into 22 segments. Taking the two best-performing and the two worst-performing sectors as examples, it can be seen clearly that the drivers of change in the indicators are far from uniform. The petroleum and coal products sector saw the greatest improvement in RP [panel (1) in Figure 2a]. A reduction in the input of natural resources, mainly crude oil, was the driver of the improvement, as is shown by the improvement of natural resource intensity *R*. The iron and steel sector, on the other hand, performed the worst, showing a significant deterioration in all the MFIs other than FD [panels (3), (11), and (15) in Figure 2]. The RP and CU rates worsened due to an increase in the volume of the input of natural resources and a decrease in the volume of cyclical use in the iron and steel sector, which is evidenced by the decline of natural resource intensity *R* and cyclical use intensity *U*. The non-ferrous metals sector [panels (9) and (13) in Figure 2] showed the greatest improvement in the CU rates. An increase in *U* was the driver of the improvement. However, it is also important to note that this sector was the second-worst cause of deterioration in RP [panel (4) in Figure 2a]. The production supply chain for goods and services (*D*_2_) and *R* were responsible for this deterioration. In particular, *D*_2_ was shown to have a large negative influence on CU rates. Improving this factor is key to improving the MFI for non-ferrous metals. On the other hand, there are some factors, such as final demand, *y*, for which a trade-off occurred (RP improved but CU rates worsened) [panels (4), (9), and (13) in Figure 2].

Unlike sectors that use materials directly to manufacture products, the sectors using materials indirectly through the supply chain are more influenced by common economic drivers (supply chain structure and final demand). In the real estate industry, which showed the second largest improvement in RP [panel (2) in Figure 2a], final demand, *y*, was the largest driver of the improvement. In the two sectors in FD’s bottom [panels (7) and (8) in Figure 2b], the contributions of final demand, *y*, and the fixed capital formation supply chain, *D*_3_, were also significant.

### 3.3 Sectors with MFI Improvements and GHG Reductions

**Figure 3:**
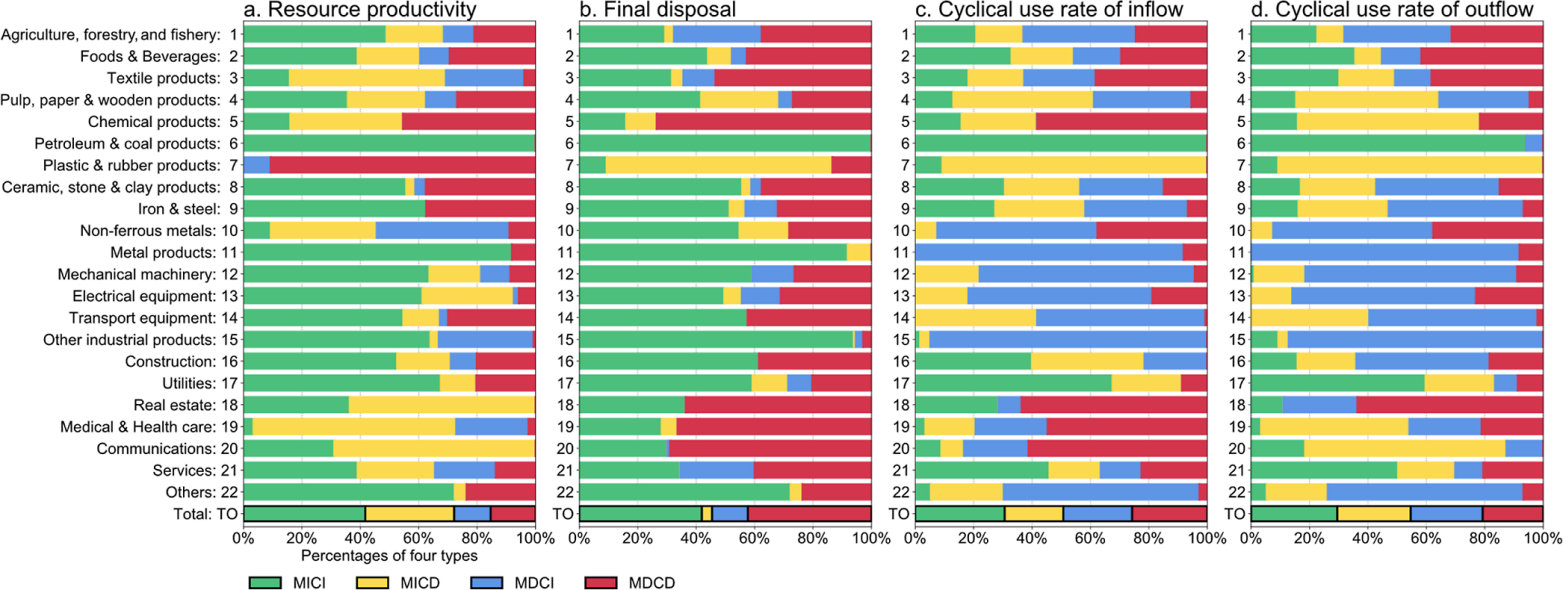
Relationship between improvements in the material flow indicators and reductions in the carbon footprint by industry. Driving forces of MFIs and CF change are classified into four types by industry: both MFIs and CF are improved, MICI; MFIs are improved, but CF is deteriorated, MICD; MFIs are deteriorated, but CF is improved, MDCI; both MFIs and CF are deteriorated, MDCD. The percentages of the 4 types in the 22 industries are weighted by the total output of the sectors included in each industry for resource productivity (a), final disposal (b), cyclical use rate of inflow (c), and cyclical use rate of outflow (d). TO: Total represents the weighted average percentage of the total of all industries.

In a number of cases, improvements in sectoral MFIs correlated with reductions in GHG. For example, for RP and FD, some sectors with improved MFIs tended to show a decrease in their CF. We classified the characteristics of the various sectors into distinct categories. Improvements in MFI (M) or reductions in CF (C) were identified as “improved” (I), while deteriorations in MFI or increases in CF were identified as “deteriorated” (D). Based on these designations, four categories were possible: MICI (i.e., MFI and CF are both “improved”), MICD, MDCI, and MDCD. Similar to Section 3.2, the 378 sectors were grouped into 22 segments. A breakdown of the output of our analysis by category is shown in Figure 3.

Petroleum and coal products (segment no. 6 in Figure 3), whose RP indicator improved the most, has an MICI (MFI and CF are both improved) ratio close to 100%; that is, in virtually all cases, both the MFI and the CF improved. However, for real estate (no. 18), which showed the second largest improvement in RP (Figure 2a), the MICI percentage was roughly 40%. This trend is similar for the segments with worsened indicators. For example, for iron and steel (no. 9), approximately 40% of the cases were classified as MDCD (MFI and CF are both deteriorated). In contrast, for non-ferrous metals (no. 10), the MDCD ratio is approximately 10%, while the MDCI (MFI deteriorated, CF improved) ratio is approximately 40%.

Some segments tended to have similar contributions to MFI improvement across all indicators, while others varied by indicators. Agriculture (no. 1), food and beverages (no. 2), petroleum and coal products (no. 6), utilities (no. 17), and services (no. 21) have similar MICI percentages. On the other hand, the manufacturing-related segments in no. 9 through no. 15 have much smaller MICI percentages in CU rates as compared to those for RP and FD. This is due to the fact that common drivers contribute to the deterioration of CU rates in these segments, as indicated by the SDA for non-ferrous metals (Figure 2c,d).

### 3.4 Incompatible Economic Drivers of Sectoral MFIs and CF Improvements

There are four drivers common to all the sectoral MFIs and CFs—the supply chain effect of direct energy inputs (Scope 1 and 2), *D*_1_; the production supply chain effects of goods and services (Scope 3; production), *D*_2_; the supply chain effect of forming fixed capital (Scope 3; fixed capital), *D*_3_; final demand structure, *y*. However, while changes in these drivers have improved MFIs, they have also contributed to a deterioration in CF in some sectors.

**Figure 4:**
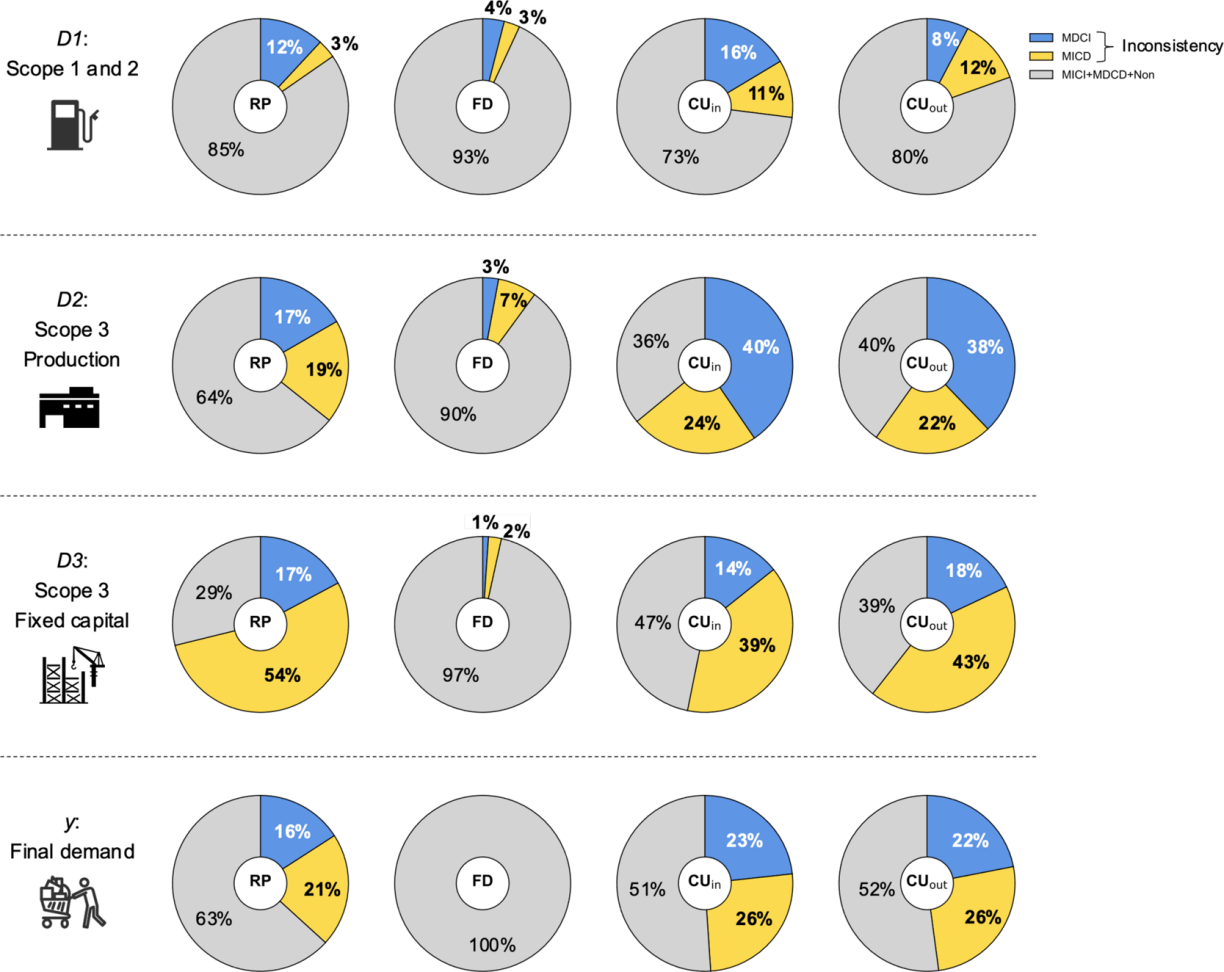
Inconsistency of improvement of material flow indicators and reduction of carbon footprint. The pie charts show the inconsistency (MDCI + MICD) of material flow indicators in each supply chain: *D*_1_, direct energy use (Scope 1 and 2); *D*_2_, goods and service production (Scope 3 production); *D*_3_, fixed capital utilization (Scope 3 fixed capital); and *y*, final demand. RP, resource productivity; FD, final disposal; CU_in_, cyclical use of inflow; CU_out_, cyclical use of outflow.

When looking at the proportion of sectors with conflicting MFI and CF changes (MDCI or MICD), it can be seen that the economic driver (*D*_1_) corresponding to Scope 1 and 2 of the supply chain shows only a small percentage (Figure 4). However, for both CU_in_ and CU_out_, more than 20% of the sectors have a conflicting contribution from the driver. In particular, waste management services have a significant increase in carbon emissions despite improvements in CU rates (see Table S5 in the Supporting Information).

As for the effect of driver *D*_2_ (Scope 3-production), the percentage of inconsistencies in the RP and FD indicators is less than 40%, while the CU rate inconsistencies exceed 60%. The percentage of MICD cases is approximately 20%, except for FD. Among the sectors with an MICD inconsistency in RP and CU rates, those with significant increases in carbon emissions are railway transport, road freight transport (except self-transport), and research and development (intra-enterprise) (see Table S5 in the Supporting Information). Driver *D*_3_ (Scope 3-fixed capital) is the only supply chain, where the inconsistency of all the indicators other than FD exceeds 50%. For RP and CU rates, the sectors with an MICD inconsistency that increased carbon emissions by more than 1 Mt/year include wholesale trade, school education (non-public institutions), and goods rental and leasing (except car rental). Unlike driver *D*_2_ of Scope 3-production, carbon emissions increased significantly in industries that indirectly use materials through fixed capital.

Only FD showed a small conflict, with less than 10% for all supply chain drivers. The RP and CU indicators had inconsistencies ranging from 15 to 71%. No indicator showed a complete win–win situation, that is, an improved MFI and a reduced CF. Among the industries included in the supply chains with significant inconsistencies in *D*_2_ and *D*_3_, those with a large material footprint can be said to be industries that not only attach a high priority to material management but also have contradictions with carbon emission reduction. Among the industries with an MF of more than 50 Mt, house rent (imputed house rent) (113 Mt of MF) and public administration (local government) (55 Mt) are in *D*_2_, while petroleum refinery products (including greases) (79 Mt) are in *D*_3_, each having MFI indicators that contradict the CF improvements (Table S5 in the Supporting Information). In addition, passenger motor vehicles (53 Mt), electricity (56 Mt), retail trade (61 Mt), and eating and drinking places (88 Mt) have CF inconsistencies in both *D*_2_ and *D*_3_.

## 4 Discussion

### 4.1 Focusing on Drivers of Change in MFIs

The four national MFIs provide a panoramic view of the material flows in Japan and have improved gradually over the past 20 years.^9^ However, focusing on further improvements in the variables that comprise these MFIs may restrict the list of policy measures capable of guiding Japan to a circular economy. For example, while RP has the advantage of relatively low data requirement and allows for comparisons between countries,^38^ focusing solely on the direct policy variables that define RP (natural resource inputs and GDP) severely limits the insights that it can provide regarding improvements in material flow. By connecting changes in the MFIs with the origin drivers of material flows, we were able to identify policy variables that indirectly dominate RP, leading to an expansion of the list of factors that could improve the indicator. Decomposition of the improvement in RP showed that the changes in household consumption and the related supply chains had a strong positive effect on the indicator, while the value-added rate had the most significant negative effect. As the value-added rate improves with an increase in wages, wage increases are not only an urgent economic policy issue for Japan^39^ but also a key strategy for improving RP.

It is also important to observe factors that are common across indicators when multiple indicators are being used. In the Japanese case, export demand led to deterioration in all the indicators. On the other hand, changes in the supply chain structure caused an improvement in RP and FD but worsened CU rates. Prioritizing measures for non-conflicting factors across indicators will avoid any unexpected adverse consequences from material flows. For example, this study showed that improving exports could improve all the MFIs without any associated conflict. For export products such as automobiles that are highly dependent on materials, introducing material efficiency strategies,^40^ including reducing yield losses or switching to alternative products, should be considered.

### 4.2 Observing the Sectoral MFIs

Economy-wide material flow is an aggregate of the individual material flows of the industries that make up a country’s economy. Hence, improvements in the material flow in individual industries can lead to better national MFIs. Our analysis of the sectoral MFIs showed that the critical drivers of change in each indicator differed by sector, implying that taking measures according to the driver characteristics of each industry is strategically sound. We found, for example, that the largest driver of the RP decline in the non-ferrous metals sector was the production supply chain. On the other hand, in the iron and steel sector, the production supply chain was the largest driver of RP improvement. Thus, lowering the intensity of natural resource use should be an effective strategic measure in this sector.

However, the absence of financial incentives for improving material flows is unlikely to accelerate voluntary efforts to improve material flow management by individual companies. Currently, global targets for materials are set in goals 8 and 12 of the Sustainable Development Goals (SDGs), yet specific norms and institutions have not been developed.^41^ Environmental, social, and governance (ESG) investment^17^ plays a role in linking the achievement of the SDGs with corporate activities.^42−44^ Hence, the CDP (carbon disclosure project) report^45^ provided to ESG investors features sections on climate change, forests, and water security but has no section on material consumption. While some companies voluntarily disclose information related to recycling and waste in response to the need for a circular economy mentioned in frameworks such as the EU taxonomy,^46^ specific targets and indicators regarding material use and inputs have not been established. Relying solely on individual companies working autonomously makes it difficult to induce a trend of managing material flows across the industry as a whole. Establishing a corporate accounting framework for material flows and setting appropriate management goals is likely needed in order to embed the principles of supply chain material management and disclosure in the practices of the business community. The design of the frameworks that disclose material-related information linked to financial aspects, similar to the task force on climate-related financial disclosure or the task force on nature-related financial disclosure, is urgently required.

### 4.3 Need to Update CU Indicators as We Move toward Carbon Neutrality

Although it is generally believed that a more circular economy is compatible with decarbonization,^9^ previous studies^47,48^ have pointed out that even with improved material recycling, the economic rebound effect may actually increase CO_2_ emissions. We have demonstrated that, in some sectors, improvements in the CU indicators did not produce CF reductions. In light of this, it is important to conduct a careful scientific investigation in order to establish whether a circular system truly contributes to decarbonization, as opposed to merely observing the CU indicators.^49^

As in the case of the CU indicators, improvements in RP were not uniformly consistent with reductions in CF. That improvements in MFIs can, at least in some cases, be inconsistent with reductions in carbon emissions is an important lesson. After 2015, national material flow indicators and GHG emissions have shown a tendency toward improvement,^9^ but we are still quite far from carbon neutrality. To make material flow management more effective in accelerating carbon mitigation, it will be necessary to understand the mechanisms behind any inconsistencies and regularly check both indicators.

### 4.4 Toward Supply Chain Management That Recognizes the Nexus of Material Consumption and GHG Emissions

In this study, we confirmed that the CU indicators are highly inconsistent with CF in the material-producing and product-manufacturing industries, while RP is highly inconsistent with CF in the services industry. Understanding the interlinkage of both material and carbon flows in these industries^16,50^ should thus be an important prerequisite to any discussion of reduction measures. In particular, the Scope 3 category showed marked conflict between improving MFIs and CF improvement. The supply chain that forms fixed capital (category 2 of Scope 3 in the GHG Protocol) tends to be inconsistent, and the material flows in this supply chain are at risk of becoming a major factor in future GHG emissions. From the perspective of carbon reduction, consideration of indicators that incorporate not only flows but also stocks^19^ will become increasingly important. An integrated assessment of material use and carbon emissions would help us chart a better path to carbon neutrality—one that avoids inconsistencies between reforming material flows and reducing carbon emissions.

## Data Availability

Data used in this study have been deposited on GitHub (http://github.com/shohata-data/mfi_sda-jp).

## Supporting Information Available

Methodological details of structural decomposition analysis; additional results; and other data (PDF)
