# Supplementary material for: Supply Chain Factors Contributing to Improved Material Flow Indicators but Increased Carbon Footprint
Source: Environ Sci Technol. 2023 Aug 17;57(34):12713–21. doi: 10.1021/acs.est.3c00859 (PMC10469450; doi:10.1021/acs.est.3c00859)
Supplement: Supplementary file 1 — es3c00859_si_001.pdf [file es3c00859_si_001.pdf]

Supporting Information

Supply Chain Factors Contributing to Improved Material Flow Indicators but Increased Carbon Footprint

By

Sho Hata*, Keisuke Nansai, Kenichi Nakajima

DOI: 10.1021/acs.est.3c00859

Contents

- Structural decomposition analysis (SDA) of material flow indicators (MFIs)
- Random mirror-image SDA

- Supplementary Figure S1: Relative difference from average of 100 mirror-image pairs: a. Resource productivity
- Supplementary Figure S2: Relative difference from average of 100 mirror-image pairs: b. Final disposal
- Supplementary Figure 3: Relative difference from average of 100 mirror-image pairs: c. Cyclical use rate of inflow
- Supplementary Figure 4: Relative difference from average of 100 mirror-image pairs: d. Cyclical use rate of outflow
- Supplementary Figure 5: Relative difference from average of 100 mirror-image pairs: e. Carbon footprint

- Supplementary Table S1: Table of symbols
- Supplementary Table S2: Category of material use intensity; natural resources and cyclical uses
- Supplementary Table S3: Driving forces of change in the material flow indicators from 2011 to 2015

- Supplementary Table S4: Inconsistency of improvement of material flow indicators and reduction of carbon footprint
- Supplementary Table S5: Industries which have a significance increase in carbon footprint (> 1 Mt) despite improvements in MFIs
- Supplementary Table S6: Industries which have a significance material footprint (> 50 Mt) with inconsistency between MFIs improvement and CF reduction in Scope 3 supply chain (D2; production, and D3; fixed capital)
- Supplementary Table S7: Sectors in the time-series input-output table and correspondence with 22 industrial segments

* Corresponding author: Sho Hata

Address: Material Cycles Division, National Institute for Environmental Studies, 16-2 Onogawa, Tsukuba, Ibaraki 305-8506, Japan

Email: hata.sho@nies.go.jp

Structural decomposition analysis (SDA) of material flow indicators (MFIs)

To conduct our SDA of MFIs, we formulated Japan's four MFIs—resource productivity (RP), final disposal (FD), cyclical use rate of inflow ($\mathrm{CU_{in}}$), and cyclical use rate of outflow ($\mathrm{CU_{out}}$)—with a capital endogenized input-output (IO) model as follows:

$$\mathrm{RP} = {}^{\mathrm{GDP}}/_{\mathrm{DMI}} = \frac{(\mathbf{v}+\mathbf{i})\mathbf{Ly}}{\mathbf{RLy}+\mathbf{O}} = \frac{\tilde{\mathbf{v}}\mathbf{Ly}}{\mathbf{RLy}+\mathbf{O}} \tag{S1}$$

$$\mathrm{FD} = \mathbf{qw}(\mathbf{RLy}) + \mathbf{q}_o\mathrm{w}_o(\mathbf{RLy}) + \mathrm{Q}_{other} \tag{S2}$$

$$\mathrm{CU_{in}} = {}^{\mathrm{CU}}/_{(\mathrm{CU}+\mathrm{DMI})} = \frac{\mathbf{ULy}}{\mathbf{ULy}+\mathbf{RLy}+\mathbf{O}} \tag{S3}$$

$$\mathrm{CU_{out}} = {}^{\mathrm{CU}}/_{\mathrm{GW}} = \frac{\mathbf{ULy}}{\mathbf{w}(\mathbf{RLy}) + \mathbf{w}_o(\mathbf{RLy}) + \mathrm{W}_{other}} \tag{S4}$$

where, $\mathbf{L} = \left(\mathbf{I}-\mathbf{A}^d\right)^{-1}$ is the Leontief invers matrix. Matrix $\mathbf{A}^d = (\mathbf{I}-\widehat{\mathbf{m}})\mathbf{A}' = (\mathbf{I}-\widehat{\mathbf{m}})\begin{pmatrix}\mathbf{A} & \mathbf{B}\\ \mathbf{C} & \mathbf{0}\end{pmatrix}$ is composed of the input coefficients including the endogenized fixed capital effects, excluding the spillover effects of imports. Matrix $\mathbf{I}$ is an identity matrix. Matrix $\mathbf{A} = (a_{ij})$ represents the input of commodity *i* into the activity of industry *j*. Matrix $\mathbf{B} = (b_{il})$, the capital formation matrix, is composed of the inputs of commodity *i* to *l* type sectors of fixed capital formation. Matrix $\mathbf{C} = (c_{lj})$, the capital utilization matrix, describes *l* types of fixed capital utilization with respect to unit production in sector *j*. Each element m_i of vector $\mathbf{m} = (m_i)$ represents the import ratio of commodity *i*. Vector $\mathbf{y} = (y_i)$ represents the final demand for commodity *i*. For domestic demand, we exclude the spillover effect of imports using vector $\mathbf{m}$. Vector $\mathbf{v} = (v_j)$ shows the amount of value-added per total output in sector *j*; matrix $\mathbf{i}$ is an identity matrix.

Matrix $\mathbf{R} = (r_{kj})$ is composed of elements r_{kj}, each of which represents the direct input of natural resources and imported products *k* per unit production in sector *j*; matrix $\mathbf{O} = (o_{kj})$ represents the direct consumption of natural resources and imported products *k* to sector *j* of final demand. Vector $\mathbf{w} = (w_i)$ represents the industrial-waste generation rate of sector

i; vector $\mathbf{w}_o = (w_{o,i})$ represents the general-waste generation rate of commodity *i*; vector $\mathbf{q} = (q_i)$ represents the final disposal rate of industrial-waste of sector *i*; and $\mathbf{q}_o = (q_{o,i})$ represents the final disposal rate of general-waste of commodity *i*. Matrix $\mathbf{U} = (u_{sj})$ shows the direct input of cyclical use, *s*, per unit production in sector *j*. W_{other} and Q_{other} represent other waste generation and other final disposal, respectively.

In Eq. (S1), GDP is expressed as $(\mathbf{v} + \mathbf{i})\mathbf{Ly} = \tilde{\mathbf{v}}\mathbf{Ly}$ and equal to the sum of final demand, including fixed capital formation. Domestic material inputs (DMI) refers to inputs of natural resources and imported products, described as the sum of the material footprint of natural resources and imported products $\mathbf{RLy}$ and their direct use in final demand $\mathbf{O}$. FD in Eq. (S2) represents the amount of landfilled waste and is defined as the sum of the landfilled industrial-waste $\mathbf{qw}(\mathbf{RLy})$, the landfilled general-waste $\mathbf{q}_o\mathbf{w}_o(\mathbf{RLy})$, and other final disposal Q_{other}. The amount of landfilled waste is calculated by multiplying the industrial- and general-waste generation, which is calculated by multiplying the inputs of natural resources $\mathbf{RLy}$ by the waste generation rate ($\mathbf{w}$ and $\mathbf{w}_o$), by the final disposal rate per waste ($\mathbf{q}$ and $\mathbf{q}_o$). In Eqs. (S3) and (S4), the amount of cyclical use (CU) is defined as the material footprint of cyclical use materials $\mathbf{ULy}$, and thus total material input of CU_{in} is defined as $\mathrm{CU} + \mathrm{DMI} = \mathbf{ULy} + \mathbf{RLy} + \mathbf{O}$. For $\mathrm{CU}_{\mathrm{out}}$ in Eq. (S4), the amount of generated waste (GW) is defined as the sum of the industrial-waste generation $\mathbf{w}(\mathbf{RLy})$, the general-waste generation $\mathbf{w}_o(\mathbf{RLy})$, and other waste generation W_{other}.

We also calculate the capital-embodied carbon footprint (CF) using the intensity of carbon emission $\mathbf{e}$ and the direct carbon emission from final demand $\mathbf{G}$, as follows:

$$\mathrm{CF} = \mathbf{eLy} + \mathbf{G} \tag{S5}$$

The changes in RP between adjacent years can be expressed as

$$\Delta\text{RP} = \Delta\text{RP}_{(t+1)} - \Delta\text{RP}_{(t)} = \frac{\tilde{\mathbf{v}}_{(t+1)}\mathbf{L}_{(t+1)}\mathbf{y}_{(t+1)}}{\mathbf{R}_{(t+1)}\mathbf{L}_{(t+1)}\mathbf{y}_{(t+1)} + \mathbf{O}_{(t+1)}} - \frac{\tilde{\mathbf{v}}_{(t)}\mathbf{L}_{(t)}\mathbf{y}_{(t)}}{\mathbf{R}_{(t)}\mathbf{L}_{(t)}\mathbf{y}_{(t)} + \mathbf{O}_{(t)}}$$
$$= \frac{\Delta\tilde{\mathbf{v}}\mathbf{L}_{(t+1)}\mathbf{y}_{(t+1)}}{\mathbf{R}_{(t+1)}\mathbf{L}_{(t+1)}\mathbf{y}_{(t+1)} + \mathbf{O}_{(t+1)}} + \frac{\tilde{\mathbf{v}}_{(t)}\Delta\mathbf{L}\mathbf{y}_{(t+1)}}{\mathbf{R}_{(t+1)}\Delta\mathbf{L}\mathbf{y}_{(t+1)} + \mathbf{O}_{(t+1)}} \tag{S6}$$
$$+ \frac{\tilde{\mathbf{v}}_{(t)}\mathbf{L}_{(t)}\Delta\mathbf{y}}{\mathbf{R}_{(t+1)}\mathbf{L}_{(t)}\Delta\mathbf{y} + \mathbf{O}_{(t+1)}} + \frac{\tilde{\mathbf{v}}_{(t)}\mathbf{L}_{(t)}\mathbf{y}_{(t)}}{\Delta\mathbf{R}\mathbf{L}_{(t)}\mathbf{y}_{(t)} + \mathbf{O}_{(t+1)}} + \frac{\tilde{\mathbf{v}}_{(t)}\mathbf{L}_{(t)}\mathbf{y}_{(t)}}{\mathbf{R}_{(t)}\mathbf{L}_{(t)}\mathbf{y}_{(t)} + \Delta\mathbf{O}}$$

As shown by Dietzenbacher and Los [1], this solution is not unique. In the case of n decomposition terms, there exist $n!$ decomposition forms that follow the structure shown in Eq. (S6), each including five separate terms with only one single change factor, i.e., Δ term, in each. To address this "non-uniqueness problem," Dietzenbacher and Los [1] proposed computing the average of the solutions of all the decomposition forms as the solution of the SDA. This full mean value (Eq. 7) is commonly used as the SDA solution [2–6]:

$$\Delta\text{RP} = \Delta\text{RP}_{\tilde{v}} + \Delta\text{RP}_{L} + \Delta\text{RP}_{y} + \Delta\text{RP}_{R} + \Delta\text{RP}_{O} = \bar{p}_{\tilde{v}} + \bar{p}_{L} + \bar{p}_{y} + \bar{p}_{R} + \bar{p}_{O} \tag{S7}$$

In this study, $\bar{p}_{\tilde{v}}, \bar{p}_{L}, \bar{p}_{y},$ and $\bar{p}_{R}$ were decomposed as follows in order to identify the key change drivers for each sector:

$$\Delta\text{RP} = \bar{p}_{\tilde{v}_i} + \bar{p}_{\tilde{v}_{(i)}} + \bar{p}_{D_1} + \bar{p}_{D_2} + \bar{p}_{D_3} + \bar{p}_{D_4} + \sum_{\alpha=1}^{3}\bar{p}_{y_{i,\alpha}} + \bar{p}_{y_{(i)}} + \sum_{\beta=1}^{5}\bar{p}_{R_{i,\beta}} + \bar{p}_{R_{(i)}} + \bar{p}_{O} \tag{S8}$$

where $\bar{p}_{D_1}$, $\bar{p}_{D_2}$, and $\bar{p}_{D_3}$ are the supply chain effects of sector *i*, broken down for the scope of the GHG protocol. Respectively, $\bar{p}_{D_1}$, $\bar{p}_{D_2}$, and $\bar{p}_{D_3}$ are the supply chain of direct energy inputs (Scope 1 and 2), the production supply chain of goods and services (Scope 3; Production), and the supply chain forming fixed capital (Scope 3; Fixed capital) in sector *i*. The effect of sector *i* can be calculated to extract the input of goods and services for sector *i* ($\mathbf{A}_i$ and $\mathbf{C}_i$) from matrix $\mathbf{A}'$, i.e. $\mathbf{A}' = \begin{pmatrix}\mathbf{A} & \mathbf{B}\\ \mathbf{C} & \mathbf{0}\end{pmatrix} = \begin{pmatrix}\mathbf{A}_i & \mathbf{0}\\ \mathbf{C}_i & \mathbf{0}\end{pmatrix} + \begin{pmatrix}\mathbf{A}_{(i)} & \mathbf{B}\\ \mathbf{C}_{(i)} & \mathbf{0}\end{pmatrix}$. We further subdivided $\mathbf{A}_i$ into the effects of energy-related industry inputs, fossil fuels, petroleum products, coal products, gas supply, electricity, private power generation, and steam and hot water supply, ($\bar{p}_{D_1}$) and the effects of

other industries ($\bar{p}_{D_2}$). $\bar{p}_{D_3}$ refers to the effect of the use of fixed capital $\mathbf{C}_i$ in sector *i*. $\bar{p}_{D_4}$ represents the supply chain effect for all industries except sector *i* (the effect of $\begin{pmatrix} \mathbf{A}_{(i)} & \mathbf{B} \\ \mathbf{C}_{(i)} & \mathbf{0} \end{pmatrix}$), where $\bar{p}_L = \bar{p}_{D_1} + \bar{p}_{D_2} + \bar{p}_{D_3} + \bar{p}_{D_4}$. The effect of sector *i* was extracted for $\bar{p}_{\tilde{v}}$, $\bar{p}_R$, $\bar{p}_y$ as well. In addition, $\bar{p}_R$ was decomposed into five resource types ($\alpha = 1 \dots 5$; Biomass, Fossil fuels, Metals, Non-metallic minerals, Imported products) and $\bar{p}_y$ into three final demands ($\beta = 1 \dots 3$; Household consumption, Other domestic consumption, Exports).

In the same way, the decomposition of the other MFIs and CF are described as follows:

$$\Delta \mathrm{FD} = \bar{p}_{q_i} + \bar{p}_{q_{(i)}} + \bar{p}_{w_i} + \bar{p}_{w_{(i)}} + \sum_{\beta=1}^{5} \bar{p}_{R_{i,\beta}} + \bar{p}_{R_{(i)}} + \bar{p}_{D_1} + \bar{p}_{D_2} + \bar{p}_{D_3} + \bar{p}_{D_4} + \sum_{\alpha=1}^{3} \bar{p}_{y_{i,\alpha}} + \bar{p}_{y_{(i)}} + \bar{p}_{q_{O,i}} + \bar{p}_{q_{O,(i)}} + \bar{p}_{w_{O,i}} + \bar{p}_{w_{O,(i)}} + \bar{p}_{Q_{other}} \quad \text{(S9)}$$

$$\Delta \mathrm{CU}_{in} = \sum_{\gamma=1}^{7} \bar{p}_{U_{i,\gamma}} + \bar{p}_{U_{(i)}} + \bar{p}_{D_1} + \bar{p}_{D_2} + \bar{p}_{D_3} + \bar{p}_{D_4} + \sum_{\alpha=1}^{3} \bar{p}_{y_{i,\alpha}} + \bar{p}_{y_{(i)}} + \sum_{\beta=1}^{5} \bar{p}_{R_{i,\beta}} + \bar{p}_{R_{(i)}} + \bar{p}_O \quad \text{(S10)}$$

$$\Delta \mathrm{CU}_{out} = \sum_{\gamma=1}^{7} \bar{p}_{U_{i,\gamma}} + \bar{p}_{U_{(i)}} + \bar{p}_{D_1} + \bar{p}_{D_2} + \bar{p}_{D_3} + \bar{p}_{D_4} + \sum_{\alpha=1}^{3} \bar{p}_{y_{i,\alpha}} + \bar{p}_{y_{(i)}} + \bar{p}_{w_i} + \bar{p}_{w_{(i)}} + \sum_{\beta=1}^{5} \bar{p}_{R_{i,\beta}} + \bar{p}_{R_{(i)}} + \bar{p}_{w_{O,i}} + \bar{p}_{w_{O,(i)}} + \bar{p}_{W_{other}} \quad \text{(S11)}$$

$$\Delta \mathrm{CF} = \bar{p}_{e_i} + \bar{p}_{e_{(i)}} + \bar{p}_{D_1} + \bar{p}_{D_2} + \bar{p}_{D_3} + \bar{p}_{D_4} + \sum_{\alpha=1}^{3} \bar{p}_{y_{i,\alpha}} + \bar{p}_{y_{(i)}} + \bar{p}_G \quad \text{(S12)}$$

To find the full mean value of these SDAs would require the calculation of a maximum of 27! cases, which is not realistic. Dietzenbacher and Los [1] found that the average of the two so-called polar decomposition forms could serve as an alternative to the full mean value. In addition to the Bipolar method, De Haan [7] also proposed using the average of the two mirror-image forms as an approximate mean value. These approximating methods are used as alternatives to full decomposition when the number of driving factors is large [8–11], although it has been pointed out that they fail the factor-reversal test and are not ideal [12]. However, the validation of these methods was conducted with relatively few decomposition terms (less than $n = 5$), and to our knowledge, there are no examples of using these alternative

means for decomposition terms greater than $n = 15$.

Random mirror-image SDA

To overcome this 'non-uniqueness problem' with large decomposition terms, we developed the random mirror-image method, a method for making the average of several pairs of mirror-images [7] an approximate solution of the full mean value. First, we set an assumption that the average of multiple mirror-image pairs would be closer to the full mean value than the average of a single pair of mirror-images.

To test the assumption, we calculated the average of each of the 30 pairs of randomly generated mirror-images and the average of all 30 pairs. Independently of these 30 pairs, another 100 pairs of mirror-images were generated, and the average of all 100 pairs was calculated. The relative difference between the average of all 100 pairs and the average of each of the 30 pairs of mirror-images generated earlier and the average of all 30 pairs was calculated. The results showed that the mean value from one pair of mirror-images deviated from the mean value of 100 pairs of mirror-images by 5% to 65%, while the mean value from 30 pairs deviated by less than 5%. (See Supplementary Figures 1-5) From these results, we determined that the average value from a single mirror-image may vary from pair to pair generated, and that it is desirable to use the average of multiple pairs to get closer to the full mean value. Considering PC performance, this study generated 100 pairs of random mirror images and calculated the average of 200 forms as the solution to SDA.

Supplementary Figures

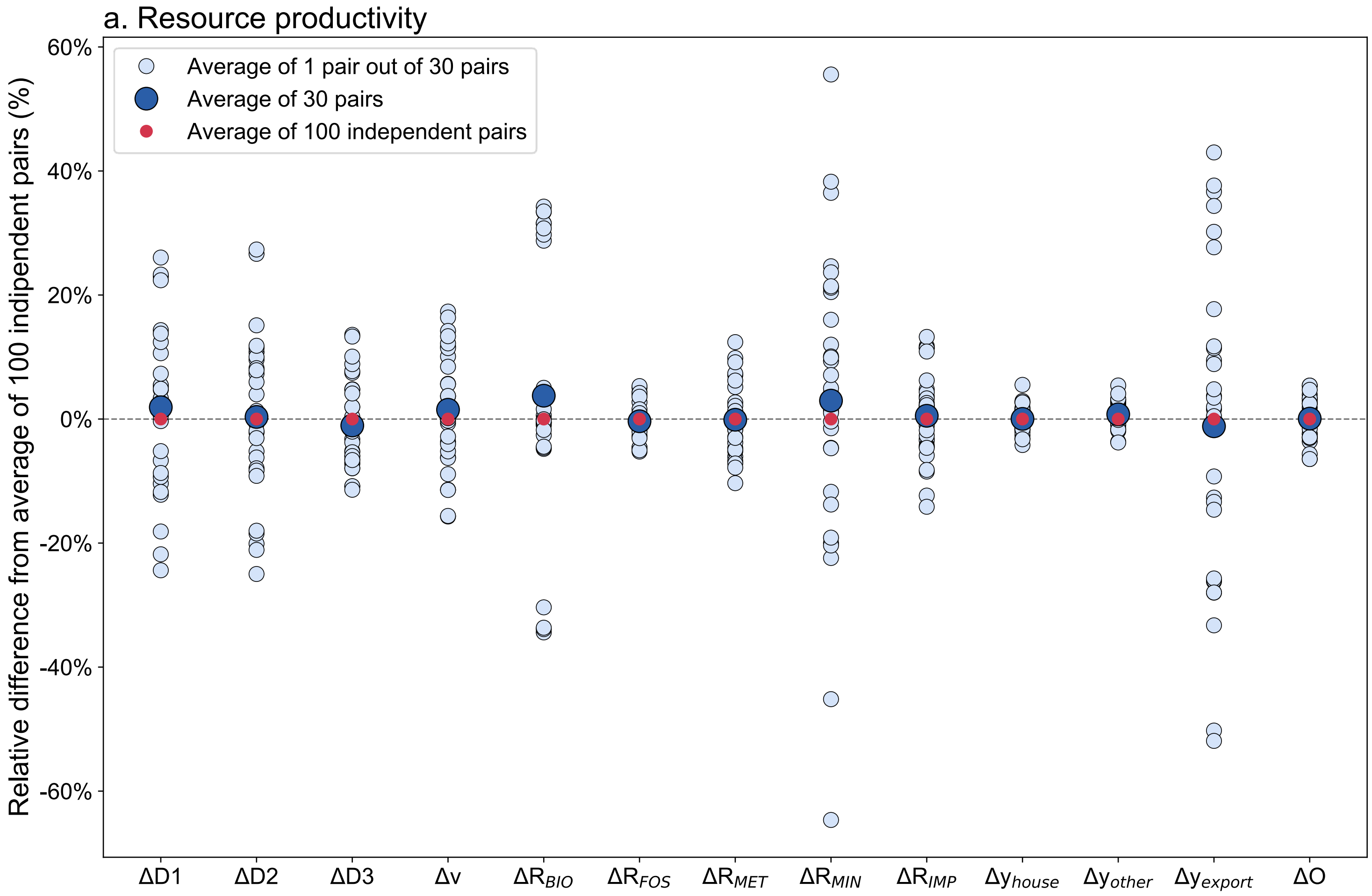

Supplementary Figure S1: Relative difference from average of 100 mirror-image pairs: a. Resource productivity

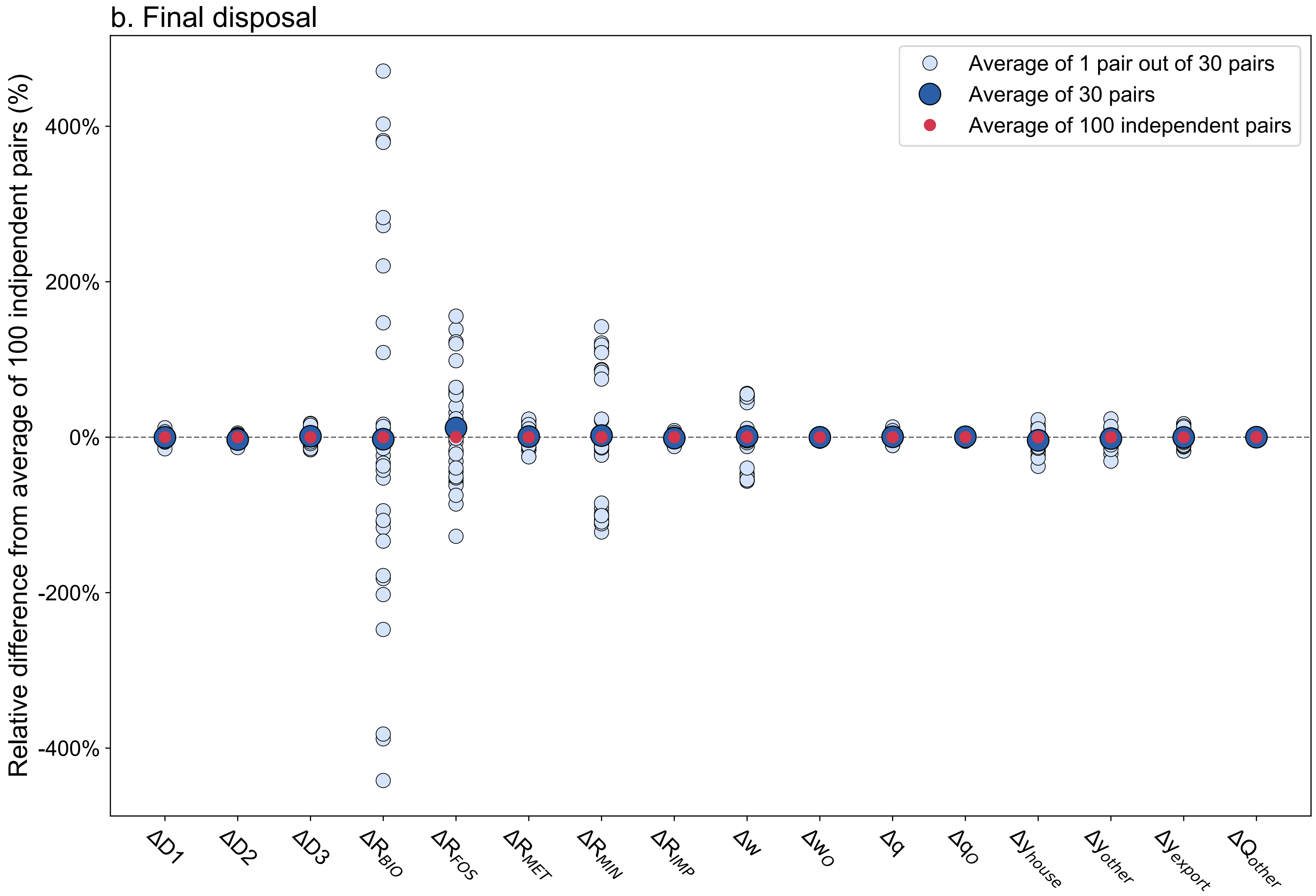

Supplementary Figure S2: Relative difference from average of 100 mirror-image pairs: b. Final disposal

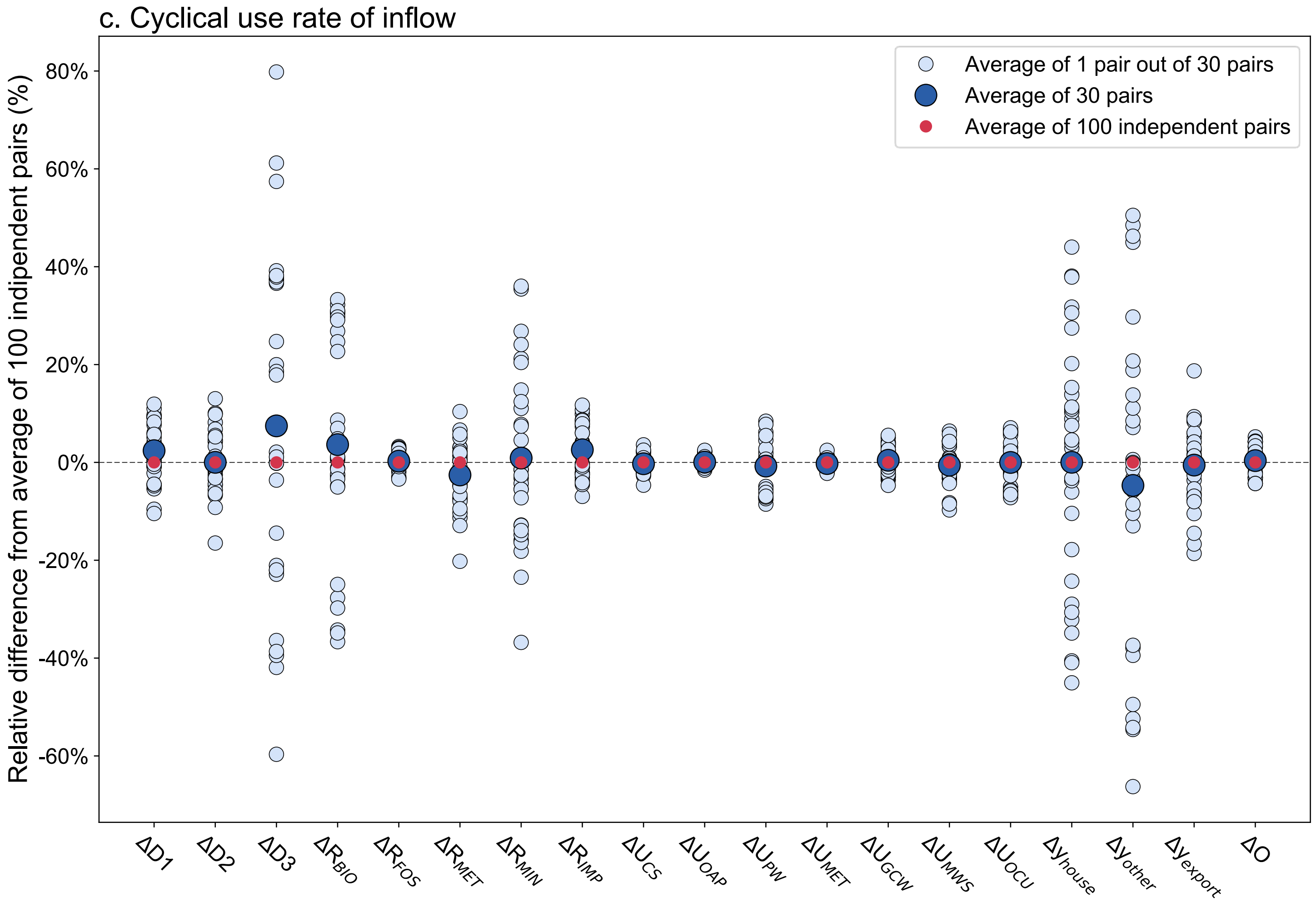

Supplementary Figure 3: Relative difference from average of 100 mirror-image pairs: c. Cyclical use rate of inflow

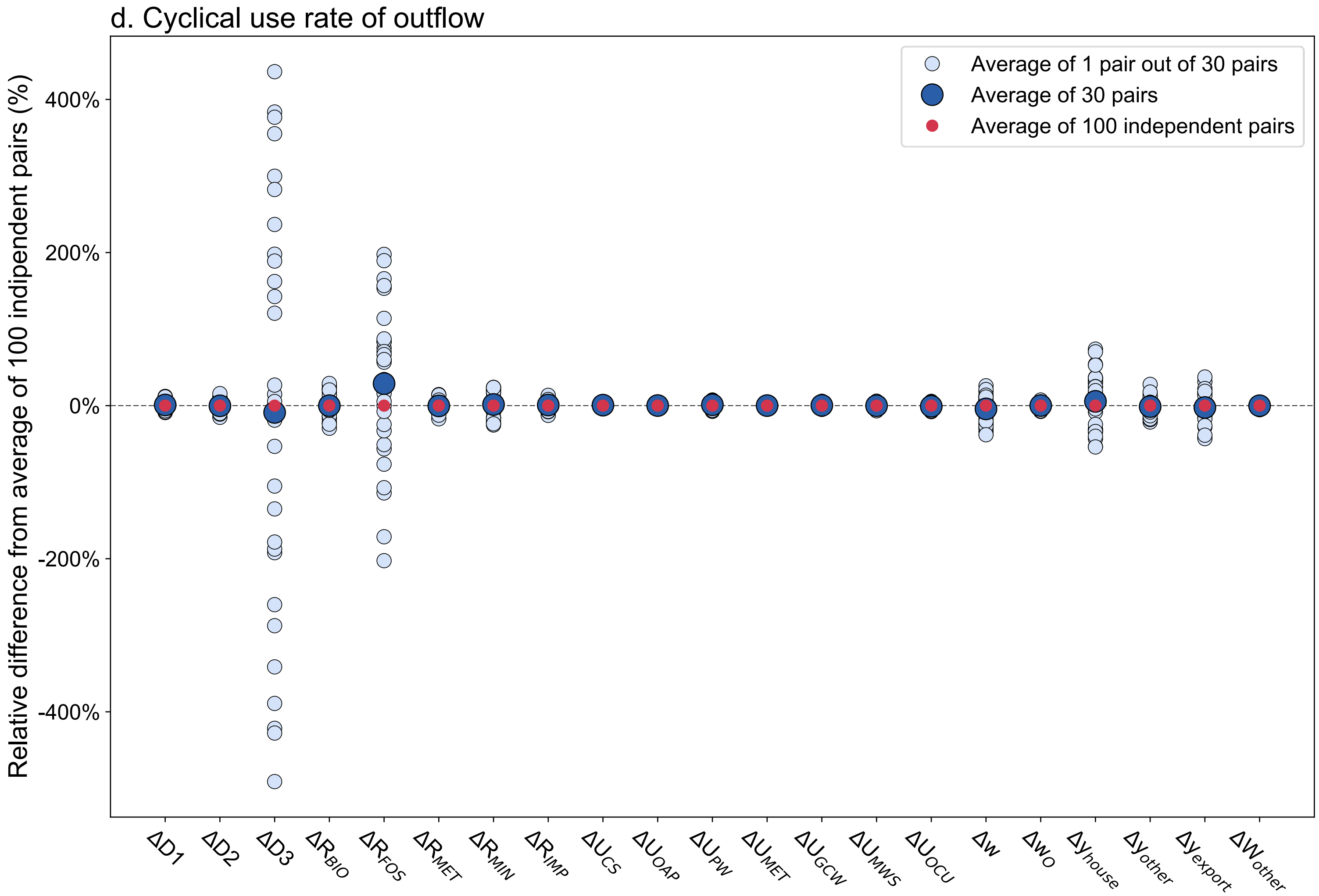

Supplementary Figure 4: Relative difference from average of 100 mirror-image pairs: d. Cyclical use rate of outflow

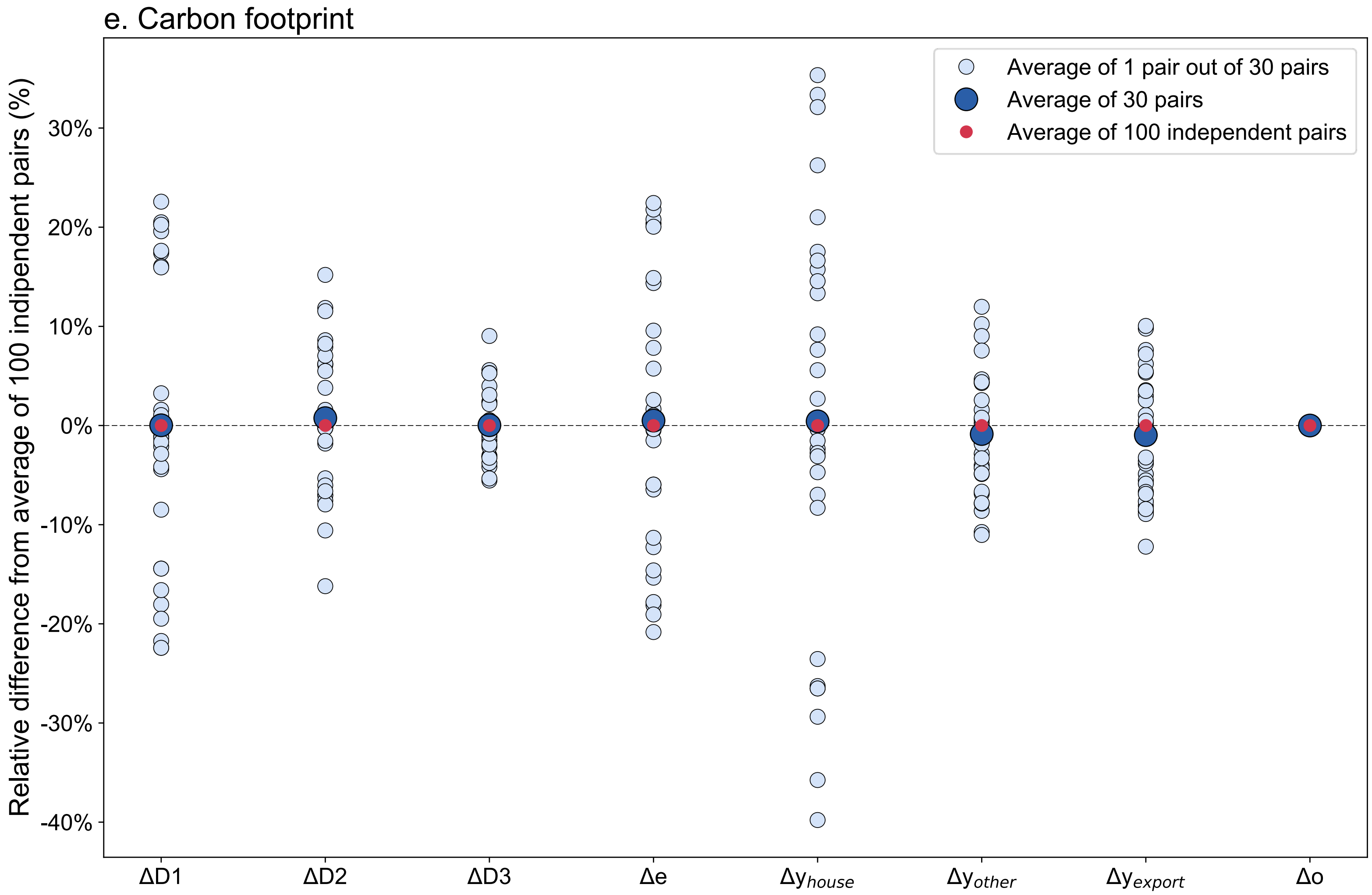

Supplementary Figure 5: Relative difference from average of 100 mirror-image pairs: e. Carbon footprint

Supplementary Tables

Table S1 Table of symbols

Symbol	Description
$\mathbf{L}$	Leontief inverse matrix
$\mathbf{A}^d$	the input coefficients including the endogenized fixed capital effects, excluding the spillover effects of imports
$\mathbf{A}$	The input coefficients which describe the input of commodity *i* into the activity of industry *j*
$\mathbf{B}$	The capital formation matrix which describes the inputs of commodity *i* to *l* type sectors of fixed capital formation
$\mathbf{C}$	The capital utilization matrix which describes *l* types of fixed capital utilization with respect to unit production in sector *j*
$\mathbf{m}$	The import ratio of commodity *i*
$\mathbf{y}$	The final demand for commodity *i*
$\mathbf{v}$	The amount of value-added per total output in sector *j*
$\mathbf{R}$	The direct input of natural resources and imported products *k* per unit production in sector *j*
$\mathbf{O}$	The direct consumption of natural resources and imported products *k* to sector *j* of final demand
$\mathbf{w}$	The industrial-waste generation rate of sector *i*
$\mathbf{w}_o$	The municipal-waste generation rate of commodity *i*
$\mathbf{q}$	The final disposal rate of industrial-waste of sector *i*
$\mathbf{q}_o$	The final disposal rate of municipal-waste of commodity *i*
$\mathbf{U}$	The direct input of cyclical use *s* per unit production in sector *j*
W_{other}	Other waste generation
Q_{other}	Other final disposal
$\mathbf{e}$	The carbon emissions per unit production in sector *j*
$\mathbf{G}$	The direct carbon emission from sector *j* of final demand

Table S2 Category of material use intensity; natural resources and cyclical use

Major categories	Subcategories
Food biomass	Rice
Food biomass	Wheat
Food biomass	Other cereals
Food biomass	Vegetables, fruit, nuts
Food biomass	Oil seeds
Food biomass	Sugar
Food biomass	Other agricultural products nec
Food biomass	Live animals
Food biomass	Meat
Food biomass	Dairy, eggs, and honey

Food biomass	Fish and aquatic resources
Wood biomass	Board and plywood
Wood biomass	Fuel wood and charcoal
Wood biomass	Lumber and sawn wood
Wood biomass	Wood pulp, chips and waste products
Other biomass	Fertilizers
Fossil fuels	Coal
Fossil fuels	Crude oil
Fossil fuels	Refined oil
Fossil fuels	Natural gas and other gaseous hydrocarbons
Fossil fuels	Other fossil fuel products
Iron ore	Iron ores and concentrates
Other metals and ores	Copper ores and concentrates
Other metals and ores	Nickel ores and concentrates
Other metals and ores	Aluminum ores and concentrates
Other metals and ores	Lead ores and concentrates
Other metals and ores	Zinc ores and concentrates
Other metals and ores	Other metal ores and concentrates
Stone, sand and clay	Stone
Stone, sand and clay	Sand and gravel
Stone, sand and clay	Clays
Other imported resource	Cement
Other industrial minerals	Lime
Other industrial minerals	Other minerals
Major metals	Iron and steel
Major metals	Copper
Major metals	Nickel
Major metals	Aluminum
Major metals	Lead
Major metals	Zinc
Other metals and ores	Other non-ferrous metals
Other metals and ores	Gold
Other metals and ores	Other Precious and specialty metals
Other metals and ores	Metals not specified
Imported products	Imported products
Cyclical use	Cinders and shoot
Cyclical use	Sludge
Cyclical use	Waste oil
Cyclical use	Waste acid/alkali
Cyclical use	Waste plastics
Cyclical use	Paper waste
Cyclical use	Wood waste
Cyclical use	Animal and vegetable residue/ food waste/ kitchen waste

Cyclical use	Metals
Cyclical use	Glass and ceramic waste
Cyclical use	Mining waste, slag
Cyclical use	Other

Table S3 Driving forces of change in the material flow indicators from 2011 to 2015

Factor	RP (thousand yen/t/yr)	FD (Mt/yr)	CU_{in} (%)	CU_{out} (%)
D1	2,380	-0.159	0.18	0.75
D2	5,491	-0.607	-0.46	-1.47
D3	3,969	0.518	-0.06	0.02
v	-3,477	–	–	–
R_{BIO}	560	-0.006	0.02	0.36
R_{FOS}	13,149	0.018	0.47	-0.04
R_{MET}	-1,733	0.136	-0.06	-0.51
R_{MIN}	-491	0.053	-0.02	-0.59
R_{IMP}	217	-0.157	0.01	0.14
U_{CS}	–	–	0.25	0.92
U_{OAP}	–	–	0.21	0.77
U_{PW}	–	–	0.14	0.50
U_{MET}	–	–	-0.24	-0.88
U_{GCW}	–	–	0.51	1.87
U_{MWS}	–	–	-0.32	-1.18
U_{OCU}	–	–	0.47	1.75
y_{house}	11,784	0.304	0.13	0.26
y_{other}	6,627	0.092	-0.03	-0.39
y_{export}	-873	0.198	-0.11	-0.16
w	–	0.768	–	-0.60
w_O	–	-0.256	–	0.15
q	–	-3.46	–	–
q_O	–	-0.451	–	–
O	930	–	0.03	–
W_{other}	–	–	–	0.10
Q_{other}	–	-0.043	–	–

Table S4-1 Inconsistency of improvement of material flow indicators and reduction of carbon footprint; D1: Scope 1 and 2.

Type	RP	FD	CU_{in}	CU_{out}
MBCG	12%	4%	16%	8%
MGCB	3%	3%	11%	12%
MGCG	54%	62%	49%	58%
MBCB	30%	30%	23%	21%
Non	1%	1%	1%	1%

Table S4-2 Inconsistency of improvement of material flow indicators and reduction of carbon footprint; D2: Scope 3 production.

Type	RP	FD	CU_{in}	CU_{out}
MBCG	17%	3%	40%	38%
MGCB	19%	7%	24%	22%
MGCG	43%	57%	19%	22%
MBCB	21%	33%	16%	18%
Non	1%	1%	1%	1%

Table S4-3 Inconsistency of improvement of material flow indicators and reduction of carbon footprint; D3: Scope 3 fixed capital.

Type	RP	FD	CU_{in}	CU_{out}
MBCG	17%	1%	14%	18%
MGCB	54%	2%	39%	43%
MGCG	13%	29%	16%	12%
MBCB	15%	67%	30%	26%
Non	1%	1%	1%	1%

Table S4-4 Inconsistency of improvement of material flow indicators and reduction of carbon footprint; D4: Final demand.

Type	RP	FD	CU_{in}	CU_{out}
MBCG	16%	0%	23%	22%
MGCB	21%	0%	23%	26%
MGCG	31%	47%	26%	25%
MBCB	26%	47%	21%	21%

Non	6%	6%	6%	6%

Table S5 Industries which have a significance increase in carbon footprint (> 1 Mt) despite improvements in MFIs

Sector name	Supply chain	MFI	CF increase (Mt CO_2-eq)
Waste management service	D1	CU_{in}, CU_{out}	3.8
Railway transport (passengers)	D2	RP, CU_{in}	1.3
Road freight transport (except self-transport)	D2	RP,CU_{in}, CU_{out}	1.6
Research and development (intra-enterprise)	D2	RP,CU_{in}, CU_{out}	1.7
Hot rolled steel	D2	CU_{in},CU_{out}	2.9
Steel pipes and tubes	D2	CU_{in}, CU_{out}	2.1
Steel ships	D2	CU_{in},CU_{out}	1.1
Wholesale trade	D3	RP, CU_{in},CU_{out}	2.7
School education (non-public institution)	D3	RP, CU_{in},CU_{out}	2.4
Goods rental and leasing (except car rental)	D3	RP, CU_{in}	1.4
Retail trade	y	RP, CU_{out}	9.0
Financial service	y	RP, CU_{in}, CU_{out}	1.2
House rent	y	RP, CU_{out}	2.7
House rent (imputed house rent)	y	RP	5.1
Mobile telecommunication	y	RP	1.1
Public administration (local government)	y	RP	1.2
Medical service (except hospitalization)	y	RP, CU_{out}	1.2
Medical service (pharmacy dispensing)	y	RP, CU_{out}	1.7
Social welfare (non-public institution)	y	RP, CU_{in}, CU_{out}	2.7
Nursing care (except facility services)	y	RP	2.1
Eating and drinking places	y	RP, CU_{in}	3.4
Sport facility service, public gardens and amusement parks	y	RP	1.6
Passenger motor vehicles	y	CU_{in}, CU_{out}	8.5
Motor vehicle parts and accessories	y	CU_{in}, CU_{out}	1.7

Table S6 Industries which have a significance material footprint (> 50 Mt) with inconsistency between MFIs improvement and CF reduction in Scope 3 supply chain (D2; production, and D3; fixed capital)

Supply chain	Sector name	MFI	MF (Mt)

D2	Passenger motor vehicles	RP, CU_{in}, CU_{out}	53
D2	Electricity	RP, CU_{in}, CU_{out}	56
D2	Retail trade	CU_{in}, CU_{out}	61
D2	House rent (imputed house rent)	RP	113
D2	Public administration (local government)	FD	55
D2	Eating and drinking places	RP, FD, CU_{in}, CU_{out}	88
D3	Petroleum refinery products (including greases)	RP, CU_{in}, CU_{out}	79
D3	Passenger motor vehicles	RP, CU_{in}, CU_{out}	53
D3	Electricity	CU_{out}	56
D3	Retail trade	RP, FD, CU_{in}, CU_{out}	61
D3	Eating and drinking places	RP, CU_{in}, CU_{out}	88

Table S7 Sectors in the time-series input-output table and correspondence with 22 industrial segments

No.	Sector name	Segment categories
1	Rice	Agriculture, forestry and fishery
2	Wheat, barley and the like	Agriculture, forestry and fishery
3	Potatoes and sweet potatoes	Agriculture, forestry and fishery
4	Pulses	Agriculture, forestry and fishery
5	Vegetables	Agriculture, forestry and fishery
6	Fruits	Agriculture, forestry and fishery
7	Sugar crops	Agriculture, forestry and fishery
8	Crops for beverages	Agriculture, forestry and fishery
9	Miscellaneous edible crops	Agriculture, forestry and fishery
10	Feed and forage crops	Agriculture, forestry and fishery
11	Seeds and seedlings	Agriculture, forestry and fishery
12	Flowers and plants	Agriculture, forestry and fishery
13	Miscellaneous inedible crops	Agriculture, forestry and fishery
14	Dairy cattle farming	Agriculture, forestry and fishery
15	Beef cattle	Agriculture, forestry and fishery
16	Hogs	Agriculture, forestry and fishery
17	Hen eggs	Agriculture, forestry and fishery
18	Chickens	Agriculture, forestry and fishery
19	Miscellaneous livestock	Agriculture, forestry and fishery
20	Veterinary service	Services
21	Agricultural services (except veterinary service)	Services
22	Silviculture	Agriculture, forestry and fishery

23	Logs	Agriculture, forestry and fishery
24	Special forest products (including hunting)	Agriculture, forestry and fishery
25	Marine fishery	Agriculture, forestry and fishery
26	Marine aquaculture	Agriculture, forestry and fishery
27	Inland water fishery and aquaculture	Agriculture, forestry and fishery
28	Coal mining, crude petroleum and natural gas	Others
29	Gravel and quarrying	Others
30	Crushed stones	Others
31	Miscellaneous ores	Others
32	Meat	Foods & Beverages
33	Dairy farm products	Foods & Beverages
34	Miscellaneous livestock products	Foods & Beverages
35	Frozen fish and shellfish	Foods & Beverages
36	Salted, dried or smoked seafood	Foods & Beverages
37	Bottled or canned seafood	Foods & Beverages
38	Fish paste	Foods & Beverages
39	Miscellaneous processed seafood	Foods & Beverages
40	Grain milling	Foods & Beverages
41	Flour and miscellaneous grain milled products	Foods & Beverages
42	Noodles	Foods & Beverages
43	Bread	Foods & Beverages
44	Confectionery	Foods & Beverages
45	Preserved agricultural foodstuffs	Foods & Beverages
46	Sugar	Foods & Beverages
47	Starch	Foods & Beverages
48	Dextrose, syrup and isomerized sugar	Foods & Beverages
49	Animal oil and fats, vegetable oil and meal	Foods & Beverages
50	Condiments and seasonings	Foods & Beverages
51	Prepared frozen foods	Foods & Beverages
52	Retort foods	Foods & Beverages
53	Dishes, sushi and lunch boxes	Foods & Beverages
54	Miscellaneous foods	Foods & Beverages
55	Refined sake	Foods & Beverages
56	Malt liquors	Foods & Beverages
57	Whiskey and brandy	Foods & Beverages
58	Miscellaneous liquors	Foods & Beverages
59	Tea and roasted coffee	Foods & Beverages
60	Soft drinks	Foods & Beverages
61	Manufactured ice	Foods & Beverages
62	Feeds	Foods & Beverages
63	Organic fertilizers, n.e.c.	Foods & Beverages
64	Tobacco	Foods & Beverages
65	Fiber yarns	Textile products

66	Cotton and staple fiber fabrics (including fabrics of synthetic spun fibers)	Textile products
67	Silk and artificial silk fabrics (including fabrics of synthetic filament fibers)	Textile products
68	Miscellaneous fabrics	Textile products
69	Knitting fabrics	Textile products
70	Yarn and fabric dyeing and finishing (processing on commission only)	Textile products
71	Miscellaneous fabricated textile products	Textile products
72	Woven fabric apparel	Textile products
73	Knitted apparel	Textile products
74	Miscellaneous wearing apparel and clothing accessories	Textile products
75	Bedding	Textile products
76	Carpets and floor mats	Textile products
77	Miscellaneous ready-made textile products	Textile products
78	Timber	Pulp, paper & wooden products
79	Plywood, glued laminated timber	Pulp, paper & wooden products
80	Wooden chips	Pulp, paper & wooden products
81	Miscellaneous wooden products	Pulp, paper & wooden products
82	Wooden furniture	Pulp, paper & wooden products
83	Metallic furniture	Pulp, paper & wooden products
84	Wooden fixtures	Pulp, paper & wooden products
85	Miscellaneous furniture and fixtures	Pulp, paper & wooden products
86	Pulp	Pulp, paper & wooden products
87	Paper	Pulp, paper & wooden products
88	Paperboard	Pulp, paper & wooden products
89	Corrugated cardboard	Pulp, paper & wooden products
90	Coated paper and building (construction)paper	Pulp, paper & wooden products
91	Corrugated card board boxes	Pulp, paper & wooden products
92	Miscellaneous paper containers	Pulp, paper & wooden products
93	Paper textile for medical use	Pulp, paper & wooden products
94	Miscellaneous pulp, paper and processed paper products	Pulp, paper & wooden products
95	Printing, plate making and book binding	Other industrial products
96	Chemical fertilizer	Chemical products
97	Industrial soda chemicals	Chemical products
98	Inorganic pigment	Chemical products
99	Compressed gas and liquefied gas	Chemical products
100	Salt	Chemical products
101	Miscellaneous industrial inorganic chemicals	Chemical products
102	Petrochemical basic products	Chemical products
103	Petrochemical aromatic products (except synthetic resin)	Chemical products
104	Aliphatic intermediates	Chemical products

105	Cyclic intermediates, synthetic dyes and organic pigments	Chemical products
106	Synthetic rubber	Chemical products
107	Methane derivatives	Chemical products
108	Plasticizers	Chemical products
109	Miscellaneous industrial organic chemicals	Chemical products
110	Thermo-setting resins	Chemical products
111	Thermoplastics resins	Chemical products
112	Miscellaneous synthetic resins	Chemical products
113	Chemical fibers	Chemical products
114	Medicaments	Chemical products
115	Oil and fat products and surface-active agents	Chemical products
116	Cosmetics, toilet preparations and dentifrices	Chemical products
117	Paint and varnishes	Chemical products
118	Printing ink	Chemical products
119	Agricultural chemicals	Chemical products
120	Gelatin and adhesives	Chemical products
121	Photographic sensitive materials	Chemical products
122	Miscellaneous final chemical products	Chemical products
123	Petroleum refinery products (including greases)	Petroleum & coal products
124	Coal products	Petroleum & coal products
125	Paving materials	Petroleum & coal products
126	Plastic products	Plastic & rubber products
127	Tires and inner tubes	Plastic & rubber products
128	Miscellaneous rubber products	Plastic & rubber products
129	Leather footwear	Other industrial products
130	Leather tanning, leather products and fur skins (except leather footwear)	Other industrial products
131	Sheet glass and safety glass	Ceramic, stone & clay products
132	Glass fiber and glass fiber products, n.e.c.	Ceramic, stone & clay products
133	Miscellaneous glass products	Ceramic, stone & clay products
134	Cement	Ceramic, stone & clay products
135	Ready mixed concrete	Ceramic, stone & clay products
136	Cement products	Ceramic, stone & clay products
137	Pottery, china and earthenware	Ceramic, stone & clay products
138	Clay refractories	Ceramic, stone & clay products
139	Miscellaneous structural clay products	Ceramic, stone & clay products
140	Carbon and graphite products	Ceramic, stone & clay products
141	Abrasive and its products	Ceramic, stone & clay products
142	Miscellaneous ceramic, stone and clay products	Ceramic, stone & clay products
143	Pig iron	Iron & steel
144	Ferro-alloys	Iron & steel
145	Crude steel (converters)	Iron & steel
146	Crude steel (electric furnaces)	Iron & steel

147	Scrap iron	Iron & steel
148	Hot rolled steel	Iron & steel
149	Steel pipes and tubes	Iron & steel
150	Cold-finished steel	Iron & steel
151	Coated steel	Iron & steel
152	Cast and forged steel	Iron & steel
153	Cast iron pipes and tubes	Iron & steel
154	Cast and forged materials (iron)	Iron & steel
155	Iron and steel shearing and slitting	Iron & steel
156	Miscellaneous iron or steel products	Iron & steel
157	Copper	Non-ferrous metals
158	Lead and zinc (including regenerated lead)	Non-ferrous metals
159	Aluminum (including regenerated aluminum)	Non-ferrous metals
160	Miscellaneous non-ferrous metals	Non-ferrous metals
161	Non-ferrous metal scrap	Non-ferrous metals
162	Electric wires and cables	Non-ferrous metals
163	Optical fiber cables	Non-ferrous metals
164	Rolled and drawn copper and copper alloys	Non-ferrous metals
165	Rolled and drawn aluminum	Non-ferrous metals
166	Non-ferrous metal castings and forgings	Non-ferrous metals
167	Nuclear fuels	Non-ferrous metals
168	Miscellaneous non-ferrous metal products	Non-ferrous metals
169	Fabricated construction-use metal products	Metal products
170	Fabricated architectural metal products	Metal products
171	Gas and oil appliances, heating and cooking apparatus	Metal products
172	Bolts, nuts, rivets and springs	Metal products
173	Metal containers, fabricated plate and sheet metal	Metal products
174	Plumbing accessories, powder metallurgy products and tools	Metal products
175	Miscellaneous metal products	Metal products
176	Boilers	Mechanical machinery
177	Turbines	Mechanical machinery
178	Engines	Mechanical machinery
179	Pumps and compressors	Mechanical machinery
180	Conveyors	Mechanical machinery
181	Refrigerators and air conditioning apparatus	Mechanical machinery
182	Bearings	Mechanical machinery
183	Miscellaneous general-purpose machinery	Mechanical machinery
184	Machinery for agricultural use	Mechanical machinery
185	Machinery and equipment for construction and mining	Mechanical machinery
186	Textile machinery	Mechanical machinery
187	Metal machine tools	Mechanical machinery

188	Metal processing machinery	Mechanical machinery
189	Machinists' precision tools	Mechanical machinery
190	Semiconductor making equipment	Mechanical machinery
191	Metal molds	Mechanical machinery
192	Vacuum equipment and vacuum component	Mechanical machinery
193	Robots	Mechanical machinery
194	Chemical machinery	Mechanical machinery
195	Miscellaneous production machinery	Mechanical machinery
196	Copy machine	Mechanical machinery
197	Miscellaneous office machines	Mechanical machinery
198	Service industry and amusement machines	Mechanical machinery
199	Measuring instruments	Mechanical machinery
200	Medical instruments	Mechanical machinery
201	Optical instruments and lenses	Mechanical machinery
202	Ordnance	Mechanical machinery
203	Semiconductor devices	Electrical equipment
204	Integrated circuits	Electrical equipment
205	Liquid crystal panel	Electrical equipment
206	Flat-panel and electron tubes	Electrical equipment
207	Miscellaneous electronic components	Electrical equipment
208	Rotating electrical equipment	Electrical equipment
209	Transformers and reactors	Electrical equipment
210	Relay switches and switchboards	Electrical equipment
211	Wiring devices and supplies	Electrical equipment
212	Electrical equipment for internal combustion engines	Electrical equipment
213	Miscellaneous electrical devices and parts	Electrical equipment
214	Household air-conditioners	Electrical equipment
215	Household electric appliances (except air-conditioners)	Electrical equipment
216	Applied electronic equipment	Electrical equipment
217	Electric measuring instruments	Electrical equipment
218	Electric bulbs	Electrical equipment
219	Electric lighting fixtures and apparatus	Electrical equipment
220	Batteries	Electrical equipment
221	Miscellaneous electrical devices and parts	Electrical equipment
222	Wired communication equipment	Electrical equipment
223	Mobile handsets	Electrical equipment
224	Radio communication equipment (except mobile phone)	Electrical equipment
225	Radio and television sets	Electrical equipment
226	Miscellaneous communication equipment	Electrical equipment
227	Video equipment and digital camera	Electrical equipment
228	Electric audio equipment	Electrical equipment

229	Personal Computers	Electrical equipment
230	Electronic computing equipment (except personal computers)	Electrical equipment
231	Electronic computing equipment (accessory equipment)	Electrical equipment
232	Passenger motor vehicles	Transport equipment
233	Trucks, buses and miscellaneous cars	Transport equipment
234	Two-wheel motor vehicles	Transport equipment
235	Internal combustion engines for motor vehicles	Transport equipment
236	Motor vehicle parts and accessories	Transport equipment
237	Steel ships	Transport equipment
238	Miscellaneous Ships (except steel ships)	Transport equipment
239	Internal combustion engines for vessels	Transport equipment
240	Repair of ships	Transport equipment
241	Rolling stock	Transport equipment
242	Repair of rolling stock	Transport equipment
243	Aircrafts	Transport equipment
244	Repair of aircrafts	Transport equipment
245	Bicycles	Transport equipment
246	Miscellaneous transport equipment	Transport equipment
247	Toys and games	Other industrial products
248	Sporting and athletic goods	Other industrial products
249	Jewelry and adornments	Other industrial products
250	Watches and clocks	Other industrial products
251	Musical instruments	Other industrial products
252	Stationery	Other industrial products
253	"Tatami" (straw matting)and straw products	Other industrial products
254	Audio and video records, other information recording media	Other industrial products
255	Miscellaneous manufacturing products	Other industrial products
256	Residential construction (wooden)	Construction
257	Residential construction (non-wooden)	Construction
258	Non-residential construction (wooden)	Construction
259	Non-residential construction (non-wooden)	Construction
260	Repair of construction	Construction
261	Public construction of roads	Construction
262	Public construction of rivers, drainages and miscellaneous public construction	Construction
263	Agricultural public construction	Construction
264	Railway construction	Construction
265	Electric power facilities construction	Construction
266	Telecommunication facilities construction	Construction
267	Miscellaneous civil engineering and construction	Construction
268	Electricity	Utility

269	Private power generation	Utility
270	Gas supply	Utility
271	Steam and hot water supply	Utility
272	Water supply	Utility
273	Industrial water supply	Utility
274	Sewage disposal	Utility
275	Waste management services (public corporation)	Utility
276	Waste management services	Utility
277	Wholesale trade	Services
278	Retail trade	Services
279	Financial service	Services
280	Life insurance	Services
281	Non-life insurance	Services
282	Real estate agencies and managers	Real estate
283	Real estate rental service	Real estate
284	House rent	Real estate
285	House rent (imputed house rent)	Real estate
286	Railway transport (passengers)	Services
287	Railway transport (freight)	Services
288	Bus transport service	Services
289	Hired car and taxi transport	Services
290	Road freight transport (except self-transport)	Services
291	International shipping	Services
292	Coastal and inland water transport	Services
293	Harbor transport service	Services
294	Air transport	Services
295	Consigned freight forwarding	Services
296	Storage facility service	Services
297	Packing service	Services
298	Facility service for road transport	Services
299	Port and water traffic control	Services
300	Services related to water transport	Services
301	Airport and air traffic control (public corporation)	Services
302	Airport and air traffic control	Services
303	Services related to air transport	Services
304	Travel agency and miscellaneous services related to transport	Services
305	Postal services and mail delivery	Services
306	Fixed telecommunications	Communications
307	Mobile telecommunications	Communications
308	Services related to telecommunications	Communications
309	Public broadcasting	Communications

310	Private broadcasting	Communications
311	Cable broadcasting	Communications
312	Information services	Communications
313	Internet based services	Communications
314	Video picture, sound information, character information production (except newspaper or publication)	Communications
315	Newspaper	Communications
316	Publication	Communications
317	Public administration (central government)	Services
318	Public administration (local government)	Services
319	School education (public institution)	Services
320	School education (non-public institution)	Services
321	School lunch (public institution)	Services
322	School lunch (non-public institution)	Services
323	Social education (public institution)	Services
324	Social education (non-public institution)	Services
325	Miscellaneous educational and training institutions (public institution)	Services
326	Miscellaneous educational and training institutions	Services
327	Research institutes for natural science (public institution)	Services
328	Research institutes for cultural and social science (public institution)	Services
329	Research institutes for natural sciences (non-public institution)	Services
330	Research institutes for cultural and social science (non-public institution)	Services
331	Research institutes for natural sciences	Services
332	Research institutes for cultural and social science	Services
333	Research and development (intra-enterprise)	Services
334	Medical service (hospitalization)	Medical & Health care
335	Medical service (except hospitalization)	Medical & Health care
336	Medical service (dentistry)	Medical & Health care
337	Medical service (pharmacy dispensing)	Medical & Health care
338	Medical service (miscellaneous medical service)	Medical & Health care
339	Health and hygiene (public institution)	Medical & Health care
340	Health and hygiene	Medical & Health care
341	Social insurance	Medical & Health care
342	Social welfare (public institution)	Medical & Health care
343	Social welfare (non-public institution)	Medical & Health care
344	Social welfare	Medical & Health care
345	Nursing care (facility services)	Medical & Health care
346	Nursing care (except facility services)	Medical & Health care
347	Membership-based business associations	Services

348	Private non-profit institutions serving households, n.e.c.	Services
349	Goods rental and leasing (except car rental)	Services
350	Car rental and leasing	Services
351	Advertising services	Services
352	Motor vehicle maintenance services	Services
353	Machine repair services	Services
354	Judicial, financial and accounting services	Services
355	Civil engineering and construction services	Services
356	Worker dispatching services	Services
357	Building maintenance services	Services
358	Miscellaneous business services	Services
359	Hotels	Services
360	Eating and drinking places	Services
361	Cleaning	Services
362	Barber shops	Services
363	Beauty shops	Services
364	Public baths	Services
365	Miscellaneous cleaning, barber shops, beauty shops and public baths	Services
366	Movie theaters	Services
367	Performances (except movie theaters), theatrical comranies	Services
368	Stadiums and companies of bicycle, horse, motorcar and motorboat races	Services
369	Sport facility service, public gardens and amusement parks	Services
370	Amusement and recreation facilities	Services
371	Miscellaneous amusement and recreation services	Services
372	Photographic studios	Services
373	Ceremonial occasions	Services
374	Supplementary tutorial schools, instruction services for arts, culture and technical skills	Services
375	Miscellaneous repairs, n.e.c.	Services
376	Miscellaneous personal services	Services
377	Office supplies	Others
378	Activities not elsewhere classified	Others

References

(1) Dietzenbacher, E.; Los, B. Structural Decomposition Techniques: Sense and Sensitivity. *Econ. Syst. Res.* **1998**, *10* (4), 307–324. https://doi.org/10.1080/09535319800000023.

(2) Nansai, K.; Kagawa, S.; Suh, S.; Inaba, R.; Moriguchi, Y. Simple Indicator to Identify the Environmental Soundness of Growth of Consumption and Technology: "Eco-Velocity of Consumption." *Environ. Sci. Technol.* **2007**, *41* (4), 1465–1472. https://doi.org/10.1021/es0615876.

(3) Minx, J. C.; Baiocchi, G.; Peters, G. P.; Weber, C. L.; Guan, D.; Hubacek, K. A "Carbonizing Dragon": China's Fast Growing CO 2 Emissions Revisited. *Environ. Sci. Technol.* **2011**, *45* (21), 9144–9153. https://doi.org/10.1021/es201497m.

(4) Wang, Y.; Zhao, H.; Li, L.; Liu, Z.; Liang, S. Carbon Dioxide Emission Drivers for a Typical Metropolis Using Input-Output Structural Decomposition Analysis. *Energy Policy* **2013**, *58*, 312–318. https://doi.org/10.1016/j.enpol.2013.03.022.

(5) Yamakawa, A.; Peters, G. P. Structural Decomposition Analysis of Greenhouse Gas Emissions in Norway 1990-2002. *Econ. Syst. Res.* **2011**, *23* (3), 303–318. https://doi.org/10.1080/09535314.2010.549461.

(6) Shigetomi, Y.; Ohno, H.; Chapman, A.; Fujii, H.; Nansai, K.; Fukushima, Y. Clarifying Demographic Impacts on Embodied and Materially Retained Carbon toward Climate Change Mitigation. *Environ. Sci. Technol.* **2019**, *53* (24), 14123–14133. https://doi.org/10.1021/acs.est.9b02603.

(7) De Haan, M. A Structural Decomposition Analysis of Pollution in the Netherlands. *Econ. Syst. Res.* **2001**, *13* (2), 181–196. https://doi.org/10.1080/09537320120052452.

(8) Arto, I.; Dietzenbacher, E. Drivers of the Growth in Global Greenhouse Gas Emissions. *Environ. Sci. Technol.* **2014**, *48* (10), 5388–5394. https://doi.org/10.1021/es5005347.

(9) Plank, B.; Eisenmenger, N.; Schaffartzik, A.; Wiedenhofer, D. International Trade Drives Global Resource Use: A Structural Decomposition Analysis of Raw Material Consumption from 1990-2010. *Environ. Sci. Technol.* **2018**, *52* (7), 4190–4198. https://doi.org/10.1021/acs.est.7b06133.

(10) Wang, Z.; Su, B.; Xie, R.; Long, H. China's Aggregate Embodied CO2 Emission Intensity from 2007 to 2012: A Multi-Region Multiplicative Structural Decomposition Analysis. *Energy Econ.*

2020, *85*, 104568. https://doi.org/10.1016/j.eneco.2019.104568.

(11) Wang, H.; Li, X.; Tian, X.; Ma, L.; Wang, G.; Wang, X.; Wang, Z.; Wang, J.; Yue, Q. Socioeconomic Drivers of China's Resource Efficiency Improvement: A Structural Decomposition Analysis for 1997–2017. *Resour. Conserv. Recycl.* **2022**, *178* (March 2021), 106028. https://doi.org/10.1016/j.resconrec.2021.106028.

(12) Su, B.; Ang, B. W. Structural Decomposition Analysis Applied to Energy and Emissions: Some Methodological Developments. *Energy Econ.* **2012**, *34* (1), 177–188. https://doi.org/10.1016/j.eneco.2011.10.009.
